# A Population Genetics-Phylogenetics Approach to Inferring Natural Selection in Coding Sequences

**DOI:** 10.1371/journal.pgen.1002395

**Published:** 2011-12-01

**Authors:** Daniel J. Wilson, Ryan D. Hernandez, Peter Andolfatto, Molly Przeworski

**Affiliations:** 1 Department of Human Genetics and Department of Ecology and Evolution, University of Chicago, Chicago, Illinois, United States of America; 2 Department of Bioengineering and Therapeutic Sciences, University of California San Francisco, San Francisco, California, United States of America; 3 Department of Ecology and Evolutionary Biology and the Lewis-Sigler Institute for Integrative Genomics, Princeton University, Princeton, New Jersey, United States of America; 4 Howard Hughes Medical Institute, University of Chicago, Chicago, Illinois, United States of America; University of Arizona, United States of America

## Abstract

Through an analysis of polymorphism within and divergence between species, we can hope to learn about the distribution of selective effects of mutations in the genome, changes in the fitness landscape that occur over time, and the location of sites involved in key adaptations that distinguish modern-day species. We introduce a novel method for the analysis of variation in selection pressures within and between species, spatially along the genome and temporally between lineages. We model codon evolution explicitly using a joint population genetics-phylogenetics approach that we developed for the construction of multiallelic models with mutation, selection, and drift. Our approach has the advantage of performing direct inference on coding sequences, inferring ancestral states probabilistically, utilizing allele frequency information, and generalizing to multiple species. We use a Bayesian sliding window model for intragenic variation in selection coefficients that efficiently combines information across sites and captures spatial clustering within the genome. To demonstrate the utility of the method, we infer selective pressures acting in *Drosophila melanogaster* and *D. simulans* from polymorphism and divergence data for 100 X-linked coding regions.

## Author Summary

Species differ genetically, and the way in which they vary is informative about the workings of natural selection: the proportion of the genome subject to selection, the degree to which selection has conserved function versus favoring novel forms, and the location of genes responsible for evolutionarily important adaptations that explain differences in biology between the species. Individuals also vary within species, and that variation provides a snapshot of the process of evolution, a snapshot that is useful for contrasting recent versus long-term evolution and for understanding the role of mutations that are destined to be lost from the population. However, existing methods tend to use only one of these sources of information. We have developed a tool to analyze variation within and between species jointly that is able to detect fine-scale differences in the action of natural selection within genes. By applying this method to 100 genes surveyed in three species of fruit fly, we show that we can detect fine-scale variation in selection pressures within genes as well as changes between species.

## Introduction

The role of adaptation versus alternative, non-adaptive forces in shaping the diversity of life within and between species lies at the heart of many questions in biology [1]–[3]. Consequently, detecting the genetic signature of natural selection in patterns of polymorphism and divergence across multiple species has become a major goal of evolutionary biology [4], [5]. From analyses of polymorphism within and divergence between species, we hope to learn about the distribution of selection coefficients acting on mutations in the genome [e.g. 6]–[8], in particular the frequency and strength of positive selection [9]–[12], changes in the fitness landscape over time [13], and the specific sites in the genome that underlie adaptive phenotypes [14], [15].

Polymorphism and divergence offer complementary angles on the evolutionary process. The McDonald-Kreitman (MK) test [16] exploits this contrast to detect adaptation where divergence or polymorphism data alone might not allow one to do so, owing to variation in selection coefficients within a gene. If adaptive change occurs at a limited number of sites in an otherwise constrained gene, deleterious mutations might limit the relative rate of non-synonymous to synonymous substitution, *DN/DS*, to a value much less than 1, and thereby swamp the signal of adaptation. Yet an excess *DN/DS* ratio compared to the relative rate of non-synonymous to synonymous polymorphism, *PN/PS*, may still reveal a surplus of non-synonymous substitution compared to polymorphism, indicative of adaptive change. Therefore the MK test is a test of the null hypothesis, under the neutral theory [3], [17], that the odds ratio (*DN PS*)/(*DS PN*) equals one; a *DN/DS* ratio significantly greater than *PN/PS* is indicative of adaptive evolution between the two species.

Several model-based interpretations of the MK test have been proposed [10],[18],[19], of which the Poisson random field (PRF) approach is most widely used [18], [20], [21]. Rooted in diffusion theory, PRF does not in its native form model variation in selection coefficients within a gene except for a class of inviable mutants (but see [22]–[24]). Arguably, this sets a high threshold for detecting adaptive change, because the net effect of selection at variable sites must be adaptive change. If, as one might expect in a functional protein-coding gene, weakly deleterious mutations provide the backdrop to adaptive change through a significant contribution to polymorphism, they will inflate the *PN/PS* ratio, and thereby raise the threshold that the *DN/DS* ratio must exceed for adaptation to be detected [19], [25], [26]. Perhaps this explains in part why scans of the human or yeast genome have not found a clear excess of genes that evolve under positive directional selection compared to what is expected by chance [21], [27], [28]. The mathematical conveniences of diffusion theory, particularly the infinite sites model of mutation, make PRF simple and attractive to use. But they also make it difficult to extend to scenarios requiring multiple alleles, multiple species, sophisticated mutation models, probabilistic inference of ancestral states and variable selection pressures. Methods to detect fine-scale variation in selection pressures such as codeml [29], [30] and omegaMap [31] exist but exploit respectively divergence and polymorphism data alone.

The aim of this paper is to develop a method for directly analyzing coding sequence data within and between species in order to (i) infer the distribution of selection coefficients within species (ii) contrast that distribution between species (iii) detect variation in selection coefficients within genes. There are two main novel aspects to the method. First, we develop a combined population genetics-phylogenetics model of codon evolution that predicts patterns of polymorphism within species and divergence between species (Figure S1). Second, we use a Bayesian sliding window approach [31], [32] to model intragenic variation in selection coefficients. We demonstrate our approach with an analysis of 100 X-linked coding regions surveyed in *Drosophila melanogaster* and *D. simulans*, using *D. yakuba* as an outgroup [33].

The key parameter of the model is the population-scaled selection coefficient, *γ* = 2*PN_e_s*, where *P* is the ploidy (*P* = 1.5 for the *Drosophila* X chromosome), *N_e_* is the effective population size and fitness is defined relative to the ancestral allele so that *s* is the fitness advantage of any derived allele encoding an amino acid different to the ancestral allele. Assuming no dominance effect, homozygotes for the beneficial allele have fitness advantage 2*s*. Stop codons are assumed inviable. The mutation model is that of Hasegawa, Kishino and Yano [34], adapted for codons. The model parameters are the transition:transversion ratio *κ* and the population-scaled mutation rate *θ* = 2*PN_e_μ*, where *μ* is the mutation rate per generation. Over long timescales, the phylogenetic substitution rate for this population genetics model converges to that of Nielsen and Yang [29], the model underlying codeml [29]–[30], where their parameter for the *DN/DS* ratio, *ω*, is related to the population-scaled selection coefficient, *γ*, through the equation 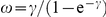 [35].

## Results

We applied our method to 100 X-linked coding regions from the fruit fly *Drosophila melanogaster* and its close relative *D. simulans*, using the *D. yakuba* reference sequence as an outgroup. Individuals were sampled from a Zimbabwean population of *D. melanogaster* and a Madagascan population of *D. simulans*, African populations that have high diversity and low linkage disequilibrium suggestive of historically large and stable population sizes [33]. The coding regions were chosen for sequencing randomly with respect to function, from the part of the X chromosome with the highest and most uniform recombination rates (as recombination rate is known to be a major determinant of diversity levels in *Drosophila*). Each region corresponds to a single exon, one per gene. The number of sequences varied across loci, with a median of 23 in *D. melanogaster* and 24 in *D. simulans*. In the following, we report the results of our analysis: the estimated distribution of fitness effects, the influence of sliding window length on what we learn about selection, examples of the local signal of variation in selection pressure, and broad patterns in the correlation in selection pressures along the genome and across evolutionary lineages.

### Inferring the Distribution of Fitness Effects

**Figure 1:**
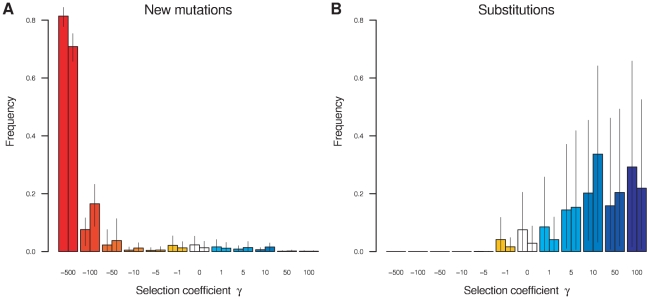
The distribution of fitness effects. The distribution of fitness effects of (A) new non-synonymous mutations and (B) amino acid substitutions in *D. melanogaster* (left bars) and *D. simulans* (right bars). The height of the bar represents the estimated frequency of each selection coefficient aggregated across codons, with the 95% credible interval indicated by a vertical line. In (A) and (B) the bars are colored according to their selection coefficient, with colors closer to red representing increasingly deleterious variants, white representing neutral variants, and colors closer to blue representing increasingly beneficial variants.

To infer the distribution of selection coefficients, also known as the distribution of fitness effects [4] (DFE), we estimated the frequency of codons at which non-synonymous mutations fall into one of twelve categories defined by the selection coefficient, *γ*. The categories encompass the range of selective effects from strongly beneficial (100, 50) through moderately beneficial (10, 5), weakly beneficial (1), neutral (0), weakly deleterious (−1), moderately deleterious (−5, −10) and strongly deleterious (−50, −100) to what is effectively inviable (−500). Classifying selection coefficients this way allowed us to estimate the relative frequencies of selection coefficients (the DFE) without making assumptions about the shape of the distribution. We estimated the DFE independently for each of the three lineages in the unrooted phylogeny. Figure 1A shows the inferred DFE for *D. melanogaster* and *D. simulans*, color-coded by selection coefficient. We do not present the results of the analysis of selection for the *D. yakuba* lineage because it was based on a single sequence, the reference genome [36].

The DFE gives the frequency with which new non-synonymous mutations occur. For both *D. melanogaster* and *D. simulans*, the vast majority of new non-synonymous mutations (81% and 71% respectively) have strongly deleterious fitness consequences, to the extent that they are effectively inviable (*γ* = −500). Thus, most sites are essentially completely constrained in the amino acid that they encode. Mutations with less severe deleterious effects are progressively less common for *γ* = −100, −50, −10 and −5. There is an increase in the frequency of weakly selected and neutral mutations, with 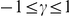 for 6.1% and 3.8% of new mutations in the two lineages respectively. Moderately beneficial mutations are less common −1.5% and 3.0% of new mutations have *γ* = 5 or 10 in the two lineages – while strongly beneficial mutations (*γ* = 50, 100) are the rarest of all with a combined frequency of 0.2% and 0.3%. Interestingly, we found that, with 99% posterior probability, at least 0.7% of newly arising non-synonymous mutations in *D. melanogaster* (and 1.9% in *D. simulans*) were moderately or strongly beneficial. The DFE is strikingly similar in the two lineages, with a slight tendency towards stronger selective effects in *D. simulans*, excluding the inviable class.

The rate at which mutations fix, relative to their neutral expectation, is given by 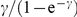. Consequently, the DFE of amino acid substitutions (Figure 1B) is enriched for beneficial mutations and greatly depleted of deleterious mutations. In both *D. melanogaster* and *D. simulans*, moderately and strongly beneficial mutations dominate the substitution process (80% and 91% of substitutions in the two lineages respectively), despite their rarity among mutations. The DFE of amino acid substitutions is similar for both lineages, albeit with a somewhat greater contribution from weakly beneficial, neutral and weakly deleterious mutations in *D. melanogaster*.

**Figure 2:**
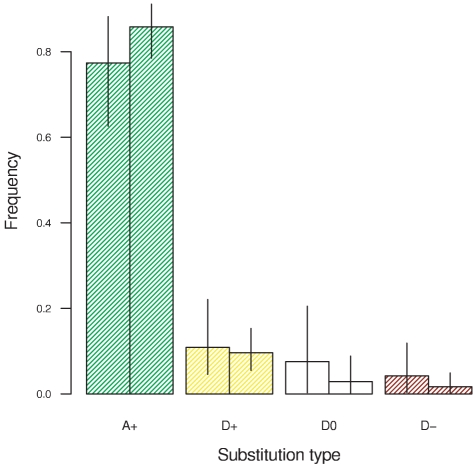
The frequency of amino acid substitutions attributable to positive selection in the *D. melanogaster* lineage (left bars) and the *D. simulans* lineage (right bars). A+: beneficial substitutions (*γ*>0) attributable to selection. D+: beneficial substitutions (*γ*>0) attributable to drift. D0: neutral substitutions (*γ* = 0) attributable to drift. D–: deleterious substitutions (*γ*<0) attributable to drift.

Smith and Eyre-Walker [10] classified amino acid substitutions into neutral substitutions expected under drift (which we label D0) and an excess of beneficial mutations driven by positive selection (which we label A+), assuming that deleterious mutations cannot fix and beneficial mutations contribute negligibly to polymorphism. Since we relax those assumptions, we can break down substitutions further into a class of beneficial mutations that would have fixed merely by drift (D+) and a class of deleterious mutations that fixed in spite of selection (D–). Figure 2 shows the frequency of each type of substitution. The vast majority of substitutions −77% in *D. melanogaster* and 86% in *D. simulans* – were beneficial and driven by selection. This finding corresponds well to estimates obtained by other methods for these two lineages [33]. In total 88% and 95% of substitutions were beneficial and driven by drift or selection. Just 4.2% and 1.7% of substitutions were deleterious, as expected almost all weakly so (*γ* = −1).

Other parameters shared across genes are reported in Table 1. To account for variation in synonymous diversity between loci, we fitted a log-normal distribution to the population-scaled mutation rates *θ* with parameters *μ_θ_* and *σ_θ_*. The estimates of these parameters yield a mean of *θ* = 31.7 per kilobase and a standard deviation of 13.2. The estimated branch length, *T*, was considerably longer for *D. melanogaster* than *D. simulans* (3.60 versus 1.48 *PN_e_* generations). Assuming the same generation length and mutation rate per generation, this suggests the *D. simulans* population has been larger on average than the *D. melanogaster* population since they split, which is consistent with the propensity towards stronger selection in the DFE. The transition:transversion ratio *κ* was similar in *D. melanogaster* and *D. simulans* (2.66 and 2.38 respectively).

**Table 1:** Parameter estimates.

	Estimate	95% credible interval
*μ_θ_*	−2.43	−2.50, −2.37
*σ_θ_*	0.400	0.358, 0.445
*T_mel_*	3.60	3.27, 3.96
*T_sim_*	1.48	1.30, 1.67
*κ_mel_*	2.66	2.45, 2.89
*κ_sim_*	2.38	2.21, 2.55
*p_mel_*	0.0105	0.00583, 0.0172
*p_sim_*	0.0277	0.0171, 0.0412

A smoothing parameter, *p*, for intragenic variation in selection coefficients was estimated independently for each lineage. The inverse of mean window length, *p* was estimated to be 0.0105 in *D. melanogaster* and 0.0277 in *D. simulans*, which corresponds to mean window lengths of 96 and 36 codons respectively. This difference may reflect the response of the smoothing parameter to the larger number of polymorphic sites in *D. simulans*, which means there is more information available. The inferred DFE is influenced somewhat by the sliding window length, and this is illustrated in Figure S2. In the extreme cases that *p* = 1 and *p* = 0, windows correspond to single codons or whole genes respectively; we refer to these two models as sitewise and genewise. Under the sitewise model, we tend to infer weaker selection in the DFE of non-synonymous mutations and amino acid substitutions. The DFE under the genewise model is rather more similar to the sliding window model, except there is an even greater frequency of effectively inviable mutations (*γ* = −500). The proportion of substitutions that were beneficial and driven by positive selection (the A+ class) is robust to window length, but under the sitewise model, there is a smaller fraction of neutral and deleterious mutations driven by drift (the D0 and D– classes). As the 95% credible intervals for the smoothing parameters excluded *p* = 1 and *p* = 0 for both *D. melanogaster* and *D. simulans*, we can conclude that the data support the sliding window model over both the sitewise and genewise models.

While our model does not account for linkage disequilibrium and demographic change, these are known to have shaped patterns of genetic diversity in *D. melanogaster* and *D. simulans* (e.g., [33], [37]), and can influence the inference of selection from allele frequency information [8], [38], [39]. Text S6 reports the results of simulations [40] that we performed to investigate the effects of these forces using demographic scenarios and recombination rates estimated for *Drosophila* [33], [37]. We found that the demographic changes may cause slight underestimation of the frequency of moderately beneficial mutations in *D. simulans*, but the overall effect was weak, indicating robustness to this model violation. We found that the low levels of linkage disequilibrium observed in *D. melanogaster* and *D. simulans* led to no additional bias beyond that induced by the demographic change (Figure S6).

### Localizing the Signal of Selection

**Figure 3:**
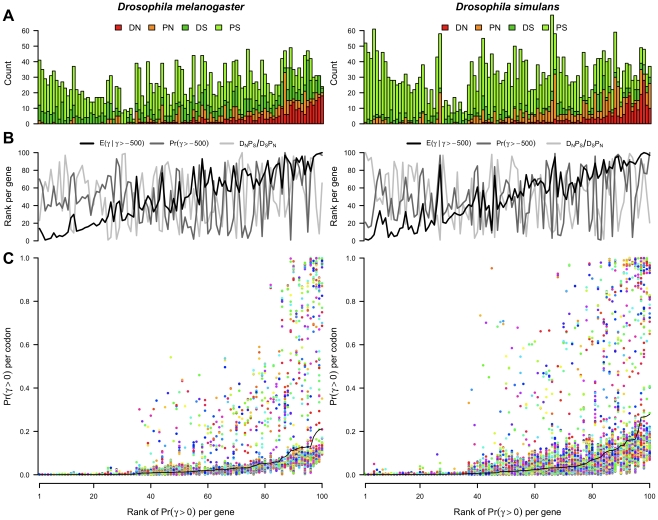
The posterior probability of positive selection across genes and codons. (A) The number of non-synonymous substitutions (DN) and polymorphisms (PN) and synonymous substitutions (DS) and polymorphisms (PS) per gene in the *D. melanogaster* and *D. simulans* lineages. (B) The rank per gene of various measures of selection. 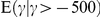: mean selection coefficient at viable sites. 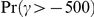: proportion of sites viable. 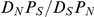: odds ratio of the McDonald-Kreitman table. (C) 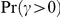, the posterior probability of positive selection per codon (points) and per gene (black line). Points are colored randomly to aid visualization. In (A), (B) and (C), genes are ordered horizontally by the rank of 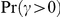 per gene.

In addition to estimating the frequency of selection coefficients across all codons (the DFE), our method yields codon-specific posterior probabilities for each selection coefficient, allowing the signal of selection to be localized. At a particular codon, there are a number of ways to summarize the distribution of selection coefficients including the probability of positive selection, the probability of viability, and the mean selection coefficient given that the codon is viable. Whole gene versions of these summary statistics can be calculated by taking the mean across codons. Figure 3 shows the evidence for positive selection across genes and sites, where genes are ordered horizontally according to the rank of the posterior probability of positive selection per gene.

Much of the variability in the evidence for positive selection at the whole gene level can be understood in terms of the entries of the McDonald-Kreitman table (Figure 3A). The ratio of the relative number of non-synonymous to synonymous substitutions (*DN/DS*), and the corresponding quantity for polymorphisms (*PN/PS*) are both strongly correlated with the probability of positive selection per gene (Spearman rank correlation coefficients of 0.81 and 0.72 respectively in *D. melanogaster*, 0.70 and 0.75 respectively in *D. simulans*). Surprisingly however, the odds ratio underlying the MK test, (*DN PS*)/(*DS PN*), was uncorrelated with the probability of positive selection (Spearman rank correlations of 0.06 in *D. melanogaster* and −0.09 in *D. simulans*). Of the three statistics summarizing the distribution of selection coefficients per gene, the largest correlation was between the probability of positive selection and the mean selection coefficient conditional on viability (Spearman rank correlations of 0.92 and 0.91 in *D. melanogaster* and *D. simulans* respectively), followed by the correlation between the mean selection coefficient conditional on viability and the probability of viability (0.15 and 0.43), and lastly between the probability of positive selection and the probability of viability (0.15 and 0.26). The relationship of these statistics and the odds ratio is shown in Figure 3B.

A comparison of the probability of positive selection at the level of the whole gene versus the individual codon (Figure 3C) suggests that positive selection is not restricted to the few genes with the strongest signal of selection; rather it has affected sites in many genes, particularly in *D. simulans*, most of which are unexceptional by whole gene metrics. By using site-specific evidence for selection, we can look for unusual signatures of selection outside the usual dichotomy of adaptation versus constraint. For example, we can detect genes with a stark contrast in intragenic selection pressures owing to the occurrence of adaptation against the backdrop of widespread constraint.

**Figure 4:**
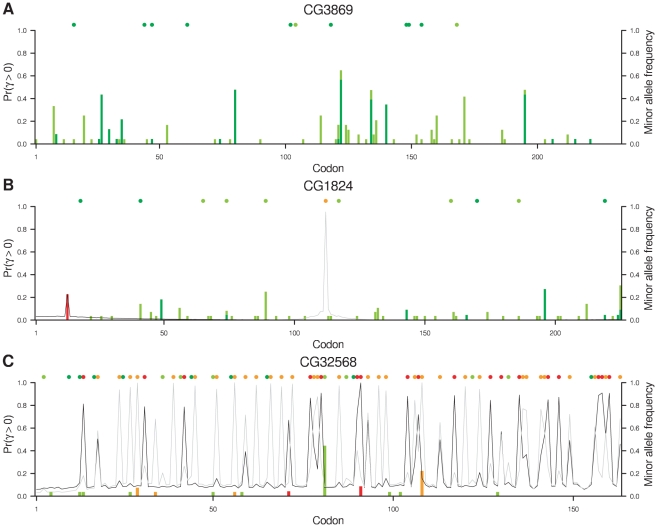
Evidence for positive selection in three genes. At each codon, the posterior probability of positive selection is plotted for *D. melanogaster* (dark grey line) and *D. simulans* (light grey line). To illustrate the signal in the data, the figure is superimposed with the sample frequency of polymorphisms in the two species (vertical bars) and substitutions along the two lineages (filled circles, above). The colors indicate synonymous variants in *D. melanogaster* (dark green) and *D. simulans* (light green) and non-synonymous variants in *D. melanogaster* (red) and *D. simulans* (orange).

On the basis of evidence at the whole gene level, protein-coding gene CG32568, of unknown function but highly expressed in adult male testes, exhibited the greatest degree of adaptation while CG3869, the ubiquitously expressed mitochondrial assembly regulatory factor Marf, exhibited the greatest degree of constraint. Based on evidence at the level of individual codons, CG1824, a ubiquitously expressed gene of unknown function, exhibited the starkest contrast in selection pressures between codons in *D. melanogaster*. Figure 4 illustrates intragenic variation in the posterior probability of positive selection for these three genes, annotated by the positions of synonymous and non-synonymous substitutions and polymorphisms. The complete absence of non-synonymous polymorphism or substitution in CG3869 (Figure 4A), in conjunction with considerable synonymous diversity, results in strong evidence against positive selection throughout the gene. CG1824 (Figure 4B) is similarly conserved for most of its length with two exceptions. A Val→Ile polymorphism in *D. melanogaster* results in a small peak in the posterior probability of positive selection at position 13, associated with a slight increase in the probability of positive selection at nearby sites owing to the sliding window model. While there is a 23% probability that this polymorphism, which coincidentally has sample frequency 23%, is positively selected, it may simply be a neutral (Pr = 31%) or deleterious (Pr = 46%) mutation that has reached appreciable frequency by drift. At position 112 there has been a Ser→His substitution in the *D. simulans* lineage that provides considerably greater evidence for the action of positive selection (Pr = 95%). Again, there is a slight increase in the probability of positive selection at nearby sites as a consequence of the sliding window model, but in the absence of other non-synonymous diversity nearby, the effect decays rapidly.

On balance, the evidence is in favor of positive selection at the non-synonymous substitution in *D. simulans* but against positive selection at the non-synonymous polymorphism in *D. melanogaster* because the former has a posterior probability greater than 50% and the latter does not. We use a 50% threshold for concluding that positive selection has acted because the prior probability is specified by the DFE that we explicitly estimated across all sites (rather than making strong prior assumptions about the relative frequency of beneficial, neutral and deleterious mutations). The fact that positively selected sites are estimated to be very rare in the DFE means that our prior probability of positive selection is very low, demanding considerable evidence to the contrary in order to surpass the threshold of 50% posterior probability.

The frequency of non-synonymous polymorphisms influences the evidence for positive selection, as illustrated by Figure S3. While the evidence for positive selection generally increases with the frequency of a derived non-synonymous mutation, in *D. melanogaster* this alone was barely sufficient to surpass a 50% probability of positive selection even with derived allele frequencies of 75% or more. In *D. simulans*, however, a non-synonymous derived allele frequency exceeding 75% provided more compelling evidence of positive selection. The reasons for these differences are multifarious and include the observation that the estimated DFE has a tendency towards stronger selection in *D. simulans*. Non-synonymous substitutions provide altogether stronger evidence for positive selection, and the large number in CG32568 in both *D. melanogaster* and *D. simulans* lineages contribute to the strong signal of adaptation (Figure 4C). Their abundance also raises the background probability of positive selection in CG32568 for both species as a result of the sliding window model. Figures S4 and S5 offer an alternative visualization of the codon-by-codon posterior distribution of selection coefficients in *D. melanogaster* and *D. simulans* respectively for CG32790, a transcription factor of unknown function that is expressed more or less ubiquitously, CG1824 and CG32568.

### Spatial and Temporal Changes in Selection Pressure

**Figure 5:**
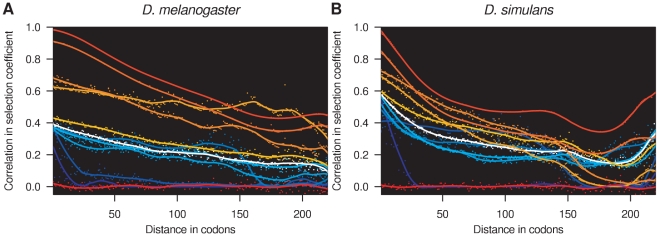
Spatial correlation in selection coefficients. Spacial correlation in selection coefficients in (A) *D. melanogaster* and (B) *D. simulans*. The correlation in the posterior probability of each selection coefficient is shown, calculated for all pairs of sites separated by the specified distance (circles). A smoothed estimate of the autocorrelation function has been superimposed (lines). The values of the selection coefficients are indicated by the coloring, which is the same as for Figure 1.

The sliding window model is designed to detect local correlation structure in selection coefficients and to infer the scale over which the selection regime varies spatially along the genome. It was found to fit the data better than either the sitewise or genewise models on the basis that the 95% credible intervals exclude *p* = 1 and *p* = 0 (Figure S2D). The influence of the sliding window model was visually apparent in the local estimates of selection coefficients within individual genes (Figure 4). Figure 5 shows the spatial correlation in the posterior distribution of selection coefficients aggregated over all genes, up to a maximum distance of 220 codons. With the exception of the inviable sites (*γ* = −500), which were assumed to occur independently of the sliding window, the posterior probability distribution of selection coefficients is highly correlated for adjacent codons. The magnitude of the spatial correlation is greatest for strongly deleterious mutations, and weakest for strongly beneficial mutations, suggesting that regions of constraint tend to be longer than regions of adaptation. As the distance between codons increases, the correlation decreases initially smoothly, and then more erratically as the number of pairs of codons involved in the calculation decreases. The spatial correlation tails off more rapidly in *D. simulans*, as expected from its shorter mean window length of 36 versus 96 codons. Even at distances of 220 codons, there is still substantial correlation in the posterior probabilities for each selection class, indicating that distant sites within the same gene are substantially more similar in selection profile than sites in different genes.

The selection coefficients in the different *Drosophila* lineages were assumed independent of one another, yet an appreciable correlation in the posterior probability distribution of selection coefficients was detectable between sites across *D. melanogaster* and *D. simulans* (Table 2). By comparing the correlation in the distribution of selection coefficients between the two species, we can examine how the selection regime has changed over evolutionary time (Figure S7). For selection coefficients *γ_mel_* and *γ_sim_*, a positive correlation in the posterior probabilities indicates an excess of sites (purple triangles). A particularly large positive correlation is seen for strongly deleterious mutations, suggesting that sites strongly constrained in one species tend to be strongly constrained in both. There is a corresponding deficit of sites strongly deleterious in one species but not the other, as evidenced by negative correlation coefficients (orange triangles). For concordant selection coefficients (both positive or both negative across species), an excess of sites was observed for which the magnitude of selection was greater in *D. simulans*, consistent with other evidence for a larger effective population size in that lineage [33]. Among discordant selection coefficients, there was a small excess of sites weakly beneficial in *D. melanogaster* yet deleterious in *D. simulans*. The cause of this pattern is unclear, but see [41] for similar observations.

**Table 2:** Correlation in selection coefficient probability between *D. melanogaster* and *D. simulans*.

*γ*	Correlation
−500	0.099
−100	0.555
−50	0.340
−10	0.038
−5	0.006
−1	0.176
0	0.233
1	0.210
5	0.197
10	0.224
50	0.182
100	0.003

## Discussion

Our method has a number of advantages over predominantly population genetics-based approaches [18], [20], [38], [39], [42], [43]. By fitting a complex, multi-parameter mutation model with repeat and back mutation, coding sequences can be directly analyzed without pooling alleles or discarding codons with more than two alleles, and discarding allele frequency information. Ancestral states are inferred probabilistically instead of by parsimony, thereby accounting for uncertainty [44]. In the analysis of polymorphism data, the advantage over phylogenetic methods [29], [30], [45]–[47] is the bottom-up model that accounts for the expected contrast between short-term and long-term evolutionary processes [16]. This is important because top-down applications of phylogenetic models to polymorphism data [31], [35] can give the misleading impression of a relaxation of functional constraint in contemporary diversity [48], [49]. In turn, the advantage of the sliding window model is that it allows inference of fine-scale variation in selection pressures by combining information across adjacent sites for statistical efficiency, but in a way that adapts to the local signal of variation in selection coefficients.

The distribution of fitness effects (DFE) is of direct interest in describing the selection regime experienced by a species. Moreover, it is important to estimate the DFE rather than making prior assumptions about its shape, as it has a strong influence on local inference of selection within genes [50]. Other methods that use allele frequency information to estimate the DFE have assumed parametric forms for the distribution, such as a gamma distribution for deleterious mutations [38], or a reflected gamma distribution [6] or normal distribution for beneficial and deleterious mutations [8]. Initial technical problems in fitting a normal and other standard distributions to the DFE by MCMC led us to switch to a discrete, non-parametric distribution defined by the relative frequency of twelve fitness classes ranging from strongly beneficial to strongly deleterious and effectively inviable. The resulting DFE estimated for the *Drosophila* coding regions looked quite unlike commonly used parametric forms (Figure 1), which may explain the difficulty in fitting. Application of the method to other datasets will determine whether the form of the DFE is a peculiarity of the *Drosophila* data or more widespread.

We made a number of simplifying assumptions in our model, amongst them that the population size is constant, that sites are independent, and that synonymous mutations are neutral. Keightley and Eyre-Walker [38], [39] and Boyko et al [6] have made advances in the co-estimation of selection and demographic change from allele frequencies. Key to their approaches is the use of computational techniques to obtain the distribution of allele frequencies when the population size changes. Presently, those techniques rely on the assumption of biallelic loci. Since the development of multiallelic models was one of our goals, a similar approach is currently out of our reach. As no method can hope to encompass all aspects of the evolutionary process, perhaps not even all the important ones, it seems reasonable to use simulations [40] in conjunction with our method to test robustness to departures from modeling assumptions. For the data analyzed in this paper, simulations suggested that demographic changes may cause slight underestimation of the frequency of moderately beneficial mutations in *D. simulans*.

The assumption of independence between sites is equivalent to assuming that sites, even adjacent sites, are completely unlinked. In fact the assumption is stronger than that since it also implies that there will be no effect of Hill-Robertson interference caused by selection acting at other loci [51]. Although the assumption of independence between sites is common in the analysis of allele frequency information [6], [8], [15], [42], [53], [38], it is of concern because selection at linked sites can skew allele frequencies at synonymous sites and may lead to false inference of selection [42]. By conducting simulations that model linkage disequilibrium [37], we were able to test the robustness of our conclusions to this assumption under recombination rates estimated for *Drosophila* [37]. Recombination rates are relatively high in the genes analyzed here. Perhaps as a result, simulations suggested that linkage did not have a large effect on our inference of the DFE. This conclusion is consistent with other investigations [8].

The classification of mutations as either non-synonymous or synonymous is a useful proxy for predicting whether mutations are likely to have a functional effect or not. However, in *Drosophila* it is well known that synonymous mutations are not strictly neutral [52]. In particular, there can be selection between codons encoding the same amino acid, thought to be attributable to differences in the efficiency of translation, mediated by the abundance of different tRNAs. The excess number of synonymous substitutions on the *D. melanogaster* lineage has been attributed to the relaxation of constraint on codon usage as a result of a reduction in the effective population size [33], implying that the difference in the branch lengths of the *D. melanogaster* and *D. simulans* lineages (Table 1) is accounted for primarily by a change in effective population size, but secondarily by the reduction in constraint on synonymous diversity in *D. melanogaster*. In the future, it may be possible to incorporate differences in the fitness of synonymous mutations into our multiallelic model.

Another simplification made during inference is to measure fitness relative to the ancestral allele. A widespread convenience common to NY98 and PRF [29], [18], measuring fitness relative to the ancestor avoids estimating selection coefficients for every possible allele, most of which go unobserved. However, it has some peculiar consequences that are often overlooked. Under positive selection (*γ*>0), the ancestral allele is always disfavored, creating a continual drive for innovation. One could characterize such a model as recurrent directional selection because, as in shift models [54], the selection regime switches upon fixation, setting up an arms race-like scenario. Under negative selection (*γ*<0), when derived alleles are disfavored, the behavior of the model is also peculiar. Were a mildly deleterious allele to fix by drift (in spite of selection), then upon fixation the selection regime would switch and rather than the back mutation restoring fitness as one might expect, it would erode it further. The convenience of models of recurrent selection has made them popular for inference and thus a natural starting point for our work. Nonetheless, it would be interesting to see what effect relaxing this assumption has on inference of selection parameters.

## Methods

### Combining Population Genetics and Phylogenetics Models

We use three steps to combine a population genetics model of the distribution of allele frequencies in a population or species with a phylogenetic model of the substitution process between species. The first step is to modify the stationary distribution of allele frequencies in the population by conditioning on the identity of the ancestral allele. Let **f** be a vector of the frequencies of *K* alleles at a site (typically, *K* = 4 nucleotides, 20 amino acids or 61 non stop codons), where 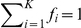. To condition the stationary distribution, 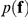, on the identity of the ancestral allele, *A*, we use Bayes' rule
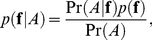
(1)where 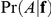 is the probability that allele *A* is ancestral given **f**, and 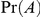 is the unconditional probability that *A* is ancestral.

The second step is to integrate over uncertainty in the population allele frequencies in order to obtain the conditional likelihood for a sample given the identity of the ancestral allele. Let **x** be a vector of the number of times each allele was observed at a particular site in a sample of size *n*, so that 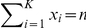. Then
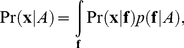
(2)where 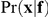 is an appropriate sampling distribution; for example the multinomial distribution when alleles are sampled at random from the population with replacement.

The third step is to sum over uncertainty in the identity of the ancestral allele of all modern populations and ancestral populations in order to calculate a joint likelihood for the observed data. On the phylogenetic tree relating our populations of interest, the tips represent modern populations that were sampled directly, and the internal nodes represent ancestral populations that were not. Felsenstein's pruning algorithm [55] makes calculation of the phylogenetic likelihood straightforward, by separating the computation into manageable chunks. The algorithm traverses the tree from tips to root, calculating 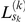, defined as the likelihood of the data observed in all populations descended from node *k*, conditional on ancestral allele *s_k_* at node *k*. For node *k* whose immediate descendants are nodes *i* and *j*,
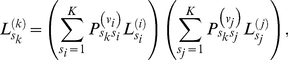
(3a)where *v_i_* is the length of the branch separating node *i* from its ancestor, and 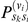 is the phylogenetic transition probability from allele *s_k_* to *s_i_* along that branch. The joint likelihood is calculated as
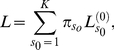
(3b)where 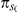 is the probability that allele *s*_0_ is ancestral. In the standard phylogenetic setting, 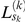 is defined at the tips to equal 1 if the sequence corresponding to that tip has allele *s_k_*, and 0 otherwise [55]. In our setting, where multiple sequences may have been sampled from the population represented by a tip, we define
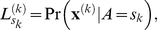
(3c)where 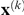 is the vector of allele sample frequencies in population *k* and the right hand formula is specified by Equation 2. Our extended pruning algorithm incorporates uncertainty in the ancestral state of modern populations at the tips of the tree. Thus it would differ from Felsenstein's algorithm even when there was a single sequence for each tip because we account for the possibility that the sequence may contain derived as well as ancestral alleles.

### Multiallelic PIMS Model

In this section we construct a combined population genetics-phylogenetics model with parent independent mutation and selection (PIMS) as the basis for an approximation to more general mutation in the next section. In parent-independent mutation, any allele can mutate to any other allele and the mutation rate is dependent only on the destination allele. The rate of mutation to allele *i* is *μ_i_* per generation.

The Wright-Dirichlet distribution is the solution to the stationary distribution of allele frequencies in a diffusion model with PIMS, assuming that fitness effects and mutation rates are small relative to the effective population size *N_e_* [56], [57]. In our notation,
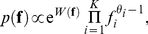
(4)where 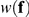 is the population fitness as a function of **f**, 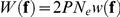 is its population-scaled counterpart, 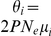 is the population-scaled rate of mutation to allele *i*, and *P* is the ploidy.

For tractability of inference and computation, we concentrate on models with two fitness classes, which we refer to as hot-or-not models. In the hot-or-not model, alleles belonging to the favored (hot) class have selective advantage *s* over other alleles; in a codon model, the two classes can be defined according to the amino acid encoded. In the hot-or-not model, the Wright-Dirichlet distribution simplifies to
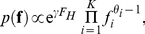
(5)where 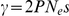 is the population-scaled selection coefficient, *F_i_* is the total frequency of alleles encoding the same amino acid as allele *i*, and *H* represents an allele belonging to the hot class.

We use the time-reversibility property to equate the probability 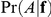 that allele *A* is ancestral to the fixation probability, which for analytic tractability we approximate as the low-mutation limit [58]

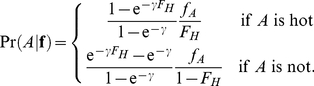
(6)We assume recurrent selection, in which the hot class comprises derived alleles encoding amino acids different to that encoded by the ancestral allele. Consequently, the sign of the population-scaled selection coefficient *γ* represents the selective advantage of mutations relative to the ancestral allele. From Equation 1,
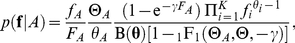
(7)where 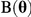 is the multivariate beta function and 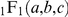 is the confluent hypergeometric function. Assuming random sampling according to the multinomial distribution we use Equation 2 to obtain the conditional likelihood
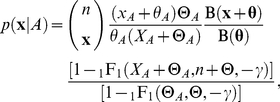
(8)where *X_A_* and Θ*_A_* are the total number of copies and total mutation rate for alleles encoding the same amino acid as the ancestral codon, and Θ is the total mutation rate across all alleles (see Text S1 for a full derivation).

The phylogenetic substitution rate specified by the population genetic model is well approximated by taking the limit that the initial frequency of a derived allele tends to zero [18], [35] so that for 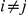,
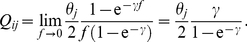
(9)The diagonal elements of the phylogenetic rate matrix are defined so that the rows sum to zero. Time is measured in units of *PN_e_* generations. At equilibrium, the allele frequencies are 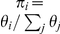; that they are independent of *γ* is a consequence of the recurrent selection model. The phylogenetic substitution matrix required by the extended pruning algorithm (Equation 3) is obtained by exponentiating the rate matrix using standard numerical techniques, so that 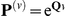.

### Multiallelic PDMS Model

In this section we utilize our PIMS model to approximate a general model of parent-dependent mutation with selection (PDMS), in which the mutation rate can differ between every pair of alleles. The approximation to PDMS that we take exploits the observations that (1) the conditional likelihood is dependent on the ancestral allele and (2) the ancestral allele will often be the genetic background upon which new mutations arise. Therefore we can modify the mutation rates in the likelihood formula (Equation 8) to suit the allelic state of the ancestral allele, re-weighting the rates to depend on the ancestral background. In Text S2 we detail the approach. Briefly, we match the rates for a parent-independent and a parent-dependent model by using average mutation probabilities, in which we calculate the expected probability of mutation from the ancestral allele *A* to every other allele, averaging over the coalescent time between two individuals in a neutral population.

We use our parent-dependent approximation to implement a codon-based analog to the HKY85 model [34]. In a codon-based HKY85 model the alleles are the *K* = 61 non stop codons, and the population-scaled mutation rate for 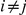 is
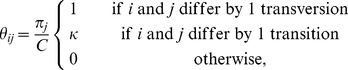
(10)where *C* normalizes the rate matrix so that the expected mutation rate is *θ*/2 per *PN_e_* generations. The diagonal elements of the matrix are defined so that the rows sum to zero. Over phylogenetic timescales, the substitution process for this population genetic model converges to the Nielsen and Yang model [29] commonly used for analyses of selection. The phylogenetic substitution process has stationary distribution *π* and (following Equation 9) rate matrix
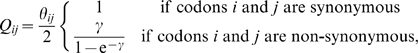
(11)where 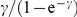 is equal to the *DN/DS* rate parameter that they call *ω*.

Owing to the approximations made in the development of likelihood functions for PIMS and PDMS models, we wished to evaluate the performance of this multiallelic selection model in a number of scenarios and over a range of parameter values. In Text S3 and Figure S8 we use simulations to examine the effect of the definition of allelic ancestry in the multiallelic setting on the accuracy of the approximate likelihood. In Text S4 and Figure S9 we test the performance of the approximate likelihood for inference over a range of parameter values: *θ* = 0.02–0.2, *κ* = 0.05–20 and *γ* drawn from a normal distribution centered on zero with a standard deviation of 10.

### Sliding Window Model for Variation in Selection Pressure

For the analysis of intragenic variation in selection pressure, we adopted a sliding window model similar to that used by omegaMap [31]. In the sliding window model of omegaMap, it is assumed that there are contiguous blocks or windows within the locus, such that all non-synonymous mutations arising within the window share the same selection coefficient. We modify this approach by allowing, with some probability, the non-synonymous mutations at any site to possess a selection coefficient different to that of the window.

We model the distribution of selection coefficients, also known as the distribution of fitness effects (DFE) [4] using a discrete range of values of *γ*. We define two classes of selection coefficient, *G*_1_ and *G*_2_, containing Γ_1_ and Γ_2_ levels of *γ* each. The first class provides values of *γ* that the window as a whole may take, and the second class provides values of *γ* that individual codons may take independently of the window within which they are situated. We specified Γ_1_ = 11, *G*_1_ = {−100, −50, −10, −5, −1, 0, 1, 5, 10, 50, 100}, which encompasses the spectrum of fitness effects from strongly deleterious, through moderately and weakly deleterious, neutral, weakly and moderately beneficial to strongly beneficial. We specified Γ_2_ = 1, *G*_2_ = {−500}, a strength of selection that corresponds effectively to inviability. The rationale for this approach was to allow individual sites within a window to be inviable, while maintaining a spatial dependency at viable sites.

The DFE is then given by the vectors *λ*_1_ and *λ*_2_, which together sum to 1. *λ*_1*i*_ is the probability that a codon takes on the selection coefficient of its window, and the window has selection coefficient *G*_1*i*_. *λ*_2*i*_ is the probability that a codon takes on a selection coefficient different to its window, and that selection coefficient is *G*_2*i*_. 
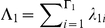
 is the total probability that a codon takes on the selection coefficient of its window. 
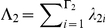
 is the total probability that a codon takes on a selection coefficient different from its window.

The length of windows is geometrically distributed and controlled by the smoothing parameter *p*, which is the probability that one window ends and another begins between a pair of adjacent codons. The average length of a window is 1/*p*. When *p* is smaller, windows are longer, which leads to greater smoothing in the estimates of variation in selection coefficients along the gene. At one extreme, *p* = 0, there is a single window per locus. Sites may be viable or inviable; those that are viable share the same selection coefficient. This “genewise” model, is equivalent to that used in the standard PRF [18], [20]. At the other extreme, *p* = 1, every codon has its own independent *γ*. This “sitewise” model features frequently in approaches based on the site frequency spectrum (although these tend to be based on nucleotides rather than codons) [*e.g.* 8], [38], [39]. Both genewise and sitewise models have been implemented in codeml [29], [30].

### Analysis of *Drosophila* X-linked Coding Sequences

We analyzed the 100 X-linked coding sequences of *Drosophila melanogaster* and *Drosophila simulans* [33]. We include the *Drosophila yakuba* reference sequence [36] in the analysis to help attribute substitutions to the *melanogaster* or *simulans* branches. Each locus corresponds to a single exon from a single gene. The average length of coding sequence per locus was 630 base pairs.

We parameterized each of the three branches of the unrooted phylogeny separately. Employing the multiallelic model (codon-based HKY85 with selection), we estimated the distribution of fitness effects *λ*, the sliding window smoothing parameter *p*, the transition:transversion ratio *κ* and the branch length *T* for each. For each locus we also estimated a branch-specific mutation rate *θ* and branch- and site-specific selection coefficients *γ*.

Our approach was Bayesian. For the DFE, we employed a symmetric Dirichlet prior with parameter *α* = 1 for the prior on *λ* = {*λ*_1_, *λ*_2_}. This distribution is equivalent to a 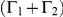-dimensional uniform distribution subject to the constraint that the elements of *λ* sum to 1. In other words, no fitness class is preferred over any other fitness class. In this sense the prior is uninformative. For the sliding window smoothing parameter *p*, we assumed a uniform distribution on the interval (0, 1). For a locus of length *L* codons, this prior gives equal probability to the number of windows between 1 and *L*. We employed improper log-uniform priors on *κ* and *T*, which are uninformative regarding the scale of the parameters in the sense that the prior probability is equal for every order of magnitude. For the branch- and locus-specific mutation rate *θ* we employed a log-normal prior distribution with mean *μ_θ_* and variance 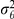 on the logarithmic scale, which allows variability in *θ* to be modeled while sharing some information across branches and loci. For the hyperparameters, we assumed an improper uniform prior on *μ* which is uninformative as to the order of magnitude of *θ*, and a log-normal prior distribution on 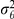 with mean 0 and variance 4 which imposes some constraint on the variability of *θ* across branches and loci in the event that the data are weakly or not informative.

We obtained a sample from the joint posterior distribution of all the parameters using Markov chain Monte Carlo (MCMC), the details of which are described in Text S5. Briefly, we ran two chains for 2,000,000 iterations each, recording the parameters at intervals of 40 iterations. After removing a burn-in of 20,000 iterations, the chains were visually compared for convergence and merged. Point estimates were calculated using the posterior mean, and 95% credible intervals were calculated as the (2.5%, 97.5%) quantiles of the posterior distribution.

### The Proportion of Amino Acid Substitutions Driven by Positive Selection

The rate of substitution, relative to neutrality, of mutations with population-scaled selection coefficient *γ* is 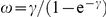. Therefore in the distribution of fitness effects of amino acid substitutions, the frequency of selection coefficient *G_i_*, where 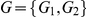 is
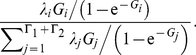
(12)


For *γ*>0, *ω* is greater than 1, so there is an excess of amino acid substitution relative to neutrality [10]. Hence for beneficial mutations we attribute a proportion 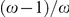 to the action of positive selection (class A+), and the remaining proportion 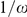, which we would have expected under neutrality, we attribute to drift (class D+). The fixation of neutral mutations is attributable to drift (class D0). Likewise, the fixation of deleterious mutations, which occurs at a lower rate than expected under neutrality, is attributable to drift acting in spite of purifying selection (class D–).

### Software

Source code and executables for the software, *gammaMap*, are available online at www.danielwilson.me.uk.

## Supporting Information

Figure S1 Combining population and phylogenetic components of an evolutionary model. At the phylogenetic timescale, fluctuations (A) in gene frequency over time are conceptually reduced (B) to a consideration of the substitution process alone. When considering a snapshot of the population (C), we employ a population genetics model of gene frequencies conditioned on the ancestral allele, whose identity is governed by the phylogenetic substitution process. To calculate the likelihood of a sample of sequences from several populations (D), we can use Felsenstein's pruning algorithm to sum over the ancestral alleles at internal nodes (d,e) as usual, and additionally at the tips (a–c). This approach accounts for the presence of derived alleles in observed molecular sequences.
(TIFF)

Figure S2 The effect of window length on the inferred distribution of fitness effects. (A) The distribution of fitness effects for new non-synonymous mutations under three models for intragenic variation in selection pressures: the sitewise, sliding window, and genewise models. (B) The distribution of fitness effects for amino acid substitutions under the three models. (C) The frequency distribution of different types of amino acid substitution. In (A), (B) and (C) frequency is represented by the vertical height of bars, with the left and right bars corresponding to the *D. melanogaster* and *D. simulans* lineages respectively. (A) and (B) employ the same color scheme for selection coefficients as Figure 1. (C) employs the same color scheme for substitution types as Figure 2. (D) The posterior density of the mean window length, in codons, for the sliding window model. The sitewise model corresponds to a fixed window length of 1 codon, and the genewise model corresponds to exactly one window per gene.
(PDF)

Figure S3 The distribution of fitness effects as a function of derived amino acid frequency in (A) *D. melanogaster* and (B) *D. simulans*. The frequency of selection coefficients was calculated in each category of sites, defined as the frequency of derived amino acids assuming a sample size of *n* = 24. Sites with *n*>24 were allocated to categories by resampling according to a hypergeometric distribution. Sites with *n*<24 were resampled according to binomial distribution. The vertical height of bars indicates the frequency of selection coefficients in that category, colored as in Figure 1. Above the barplot is printed the number of codons assigned to each category, averaged over the resampling.
(PDF)

Figure S4 The posterior probability of selection coefficients for non-synonymous mutations along three genes in the *D. melanogaster* lineage. At each codon, the height of the colored bars represents the posterior probability of the corresponding selection coefficient, where colors closer to red represent increasingly deleterious variants, white represents neutral variants, and colors closer to blue represent increasingly beneficial variants, as in Figure 1. Above the barplot are indicated the presence of synonymous (grey) and non-synonymous (black) polymorphisms (vertical lines) and substitutions (circles) in the *D. melanogaster* lineage.
(TIFF)

Figure S5 The posterior probability of selection coefficients for non-synonymous mutations along three genes in the *D. simulans* lineage. At each codon, the height of the colored bars represents the posterior probability of the corresponding selection coefficient, where colors closer to red represent increasingly deleterious variants, white represents neutral variants, and colors closer to blue represent increasingly beneficial variants, as in Figure 1. Above the barplot are indicated the presence of synonymous (grey) and non-synonymous (black) polymorphisms (vertical lines) and substitutions (circles) in the *D. simulans* lineage.
(TIFF)

Figure S6 Robustness of inference of selection coefficients to linkage and demographic change. The frequency with which sites were assigned to each of the twelve selection classes is shown separately for (A) *D. melanogaster* and (B) *D. simulans* under three simulated scenarios assuming the DFE specified by the Expected column. Scenario 1: no linkage or demographic change. Scenario 2: demographic change but no linkage. Scenario 3: linkage and demographic change.
(PDF)

Figure S7 Correlation in the posterior probability of selection coefficients between *D. melanogaster* and *D. simulans*. For each pair of selection coefficients *γ_mel_* and *γ_sim_*, the magnitude of the correlation in posterior probability across sites is indicated by the size of the triangle and the direction by its colour: purple for positive values, orange for negative values. Positive correlations indicate an excess of sites compared to the assumption of independence between lineages. Negative correlations indicate a deficit of sites. In the top right and bottom left quadrants, *γ_mel_* and *γ_sim_* are concordant (both positive or both negative respectively). These quadrants are bisected by the diagonal, which indicates trends in the strength of selection. Between the diagonal and the horizontal line at *γ_mel_* = 0, selection is stronger in *D. simulans*. Between the diagonal and the vertical line at *γ_sim_* = 0, selection is weaker in *D. simulans*. In the other two quadrants *γ_mel_* and *γ_sim_* are discordant.
(PDF)

Figure S8 The operational definition of ancestral identity affects the accuracy of the conditional gene frequency distribution. (A) When the operational definition of ancestral identity is the last allele to have fixed or – as here – the state of the population MRCA, there is a discrepancy between theory (purple bars) and simulations (green bars). Simulations, which were conducted under the codon model with *θ* = 0.3, *κ* = 1 and *γ* = 0, are in agreement with theory when the ancestral allele is common, but report an elevated probability of not sampling the ancestral allele at all, which is not predicted from theory, and could be erroneously attributed to positive selection (red bars). (B) When the operational definition of ancestral identity is the oldest allele segregating in the population, the differences are resolved. (C) The cause of the problem: an ancestral allele (cyan) is lost from the population at 3.6 *N* generations, long before one of the other alleles (purple) fixes at 6.9 *N* generations, creating appreciable periods of time when the ancestral allele is no longer segregating in the population.
(PDF)

Figure S9 Testing the multiallelic codon model by simulation. The posterior mean (circles) and 95% credible interval (vertical lines) of the mutation rate (*θ*), transition:transversion ratio (*κ*) and strength of selection (*γ*) are plotted against their true values for 200 simulated datasets under two scenarios. (A) To test the conditional allele frequency distribution (the population genetic model), inference was performed with known ancestral states. (B) To additionally test the phylogenetic model and the extended pruning algorithm, the ancestral state was recorded 10 *PN_e_* generations prior to sampling. Colored lines draw attention to datasets for which the truth lies outside the 95% credible interval. The top left number in each graph reports the number of simulations for which the 95% credible interval enveloped the truth (a range of 184–196 is desirable). In all cases 30 sequences of length 250 codons were simulated per dataset.
(PDF)

Text S1 Deriving the multiallelic hot-or-not model.
(PDF)

Text S2 Approximating parent-dependent mutation.
(PDF)

Text S3 On the definition of allelic ancestry.
(PDF)

Text S4 Testing the multiallelic model by simulation.
(PDF)

Text S5 Inference via Markov chain Monte Carlo.
(PDF)

Text S6 Robustness to linkage and demographic change.
(PDF)
