# Supplementary figures and images for: A Population Genetics-Phylogenetics Approach to Inferring Natural Selection in Coding Sequences
Source: PLoS Genet. 2011 Dec 1;7(12):e1002395. doi: 10.1371/journal.pgen.1002395 (PMC3228810; doi:10.1371/journal.pgen.1002395)

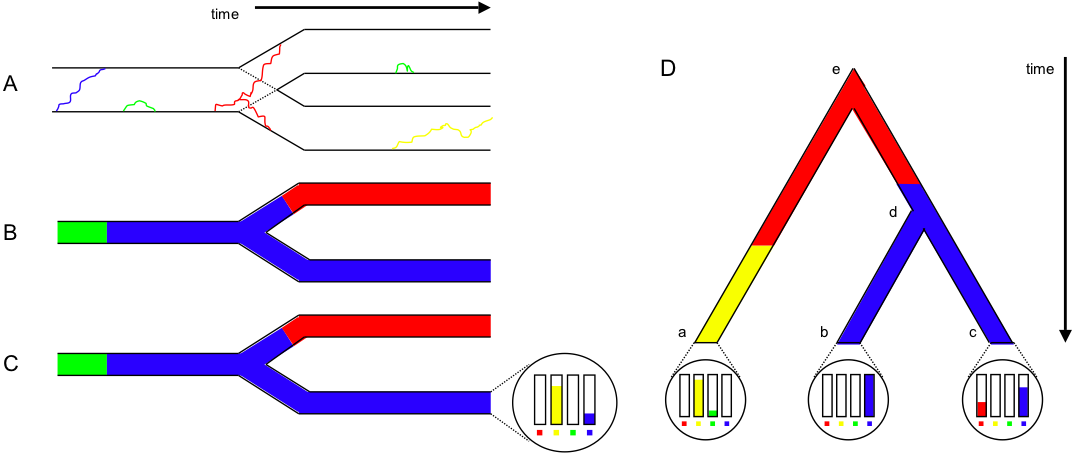

Supplement: Figure S1 — Combining population and phylogenetic components of an evolutionary model. At the phylogenetic timescale, fluctuations (A) in gene frequency over time are conceptually reduced (B) to a consideration of the substitution process alone. When considering a snapshot of the population (C), we employ a population genetics model of gene frequencies conditioned on the ancestral allele, whose identity is governed by the phylogenetic substitution process. To calculate the likelihood of a sample of sequences from several populations (D), we can use Felsenstein's pruning algorithm to sum over the ancestral alleles at internal nodes (d,e) as usual, and additionally at the tips (a–c). This approach accounts for the presence of derived alleles in observed molecular sequences. (TIFF) [file pgen.1002395.s001.tif]

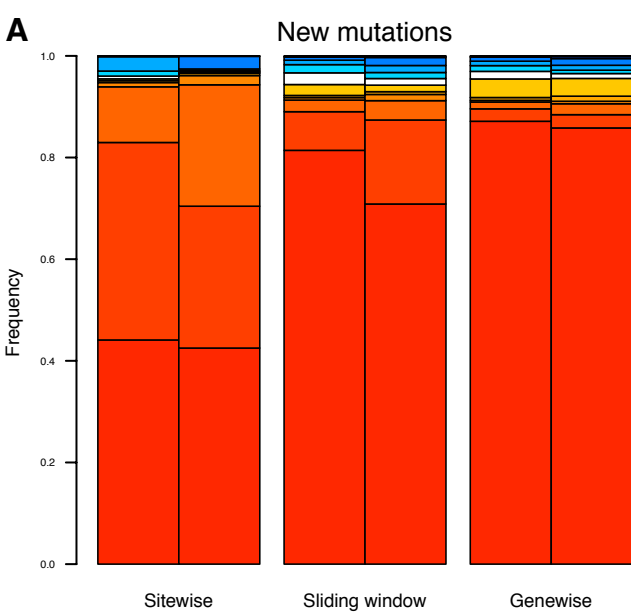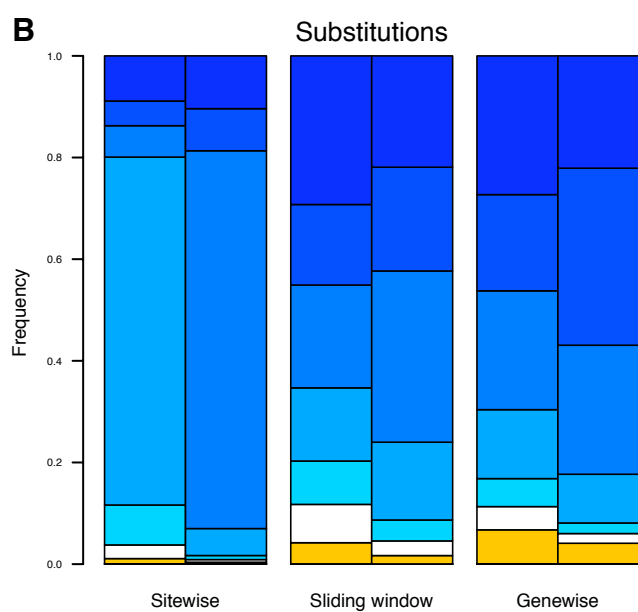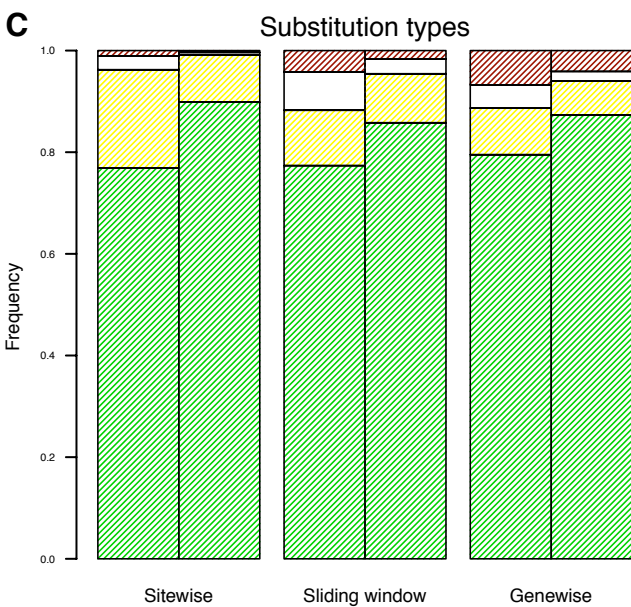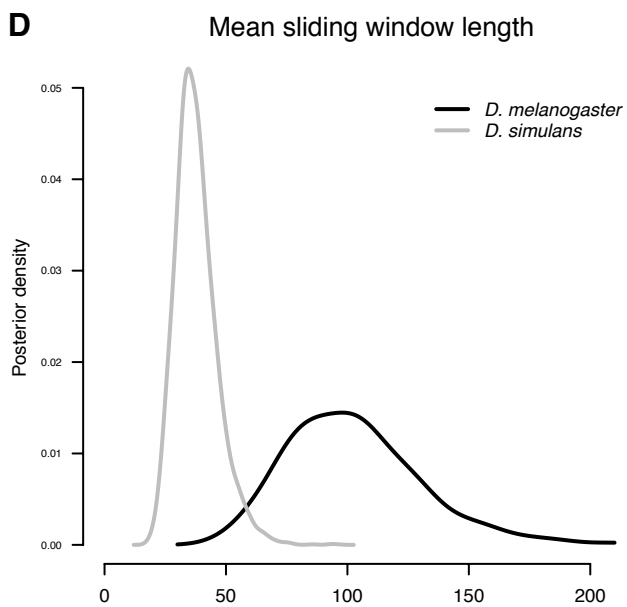

Supplement: Figure S2 — The effect of window length on the inferred distribution of fitness effects. (A) The distribution of fitness effects for new non-synonymous mutations under three models for intragenic variation in selection pressures: the sitewise, sliding window, and genewise models. (B) The distribution of fitness effects for amino acid substitutions under the three models. (C) The frequency distribution of different types of amino acid substitution. In (A), (B) and (C) frequency is represented by the vertical height of bars, with the left and right bars corresponding to the D. melanogaster and D. simulans lineages respectively. (A) and (B) employ the same color scheme for selection coefficients as Figure 1. (C) employs the same color scheme for substitution types as Figure 2. (D) The posterior density of the mean window length, in codons, for the sliding window model. The sitewise model corresponds to a fixed window length of 1 codon, and the genewise model corresponds to exactly one window per gene. (PDF) [file pgen.1002395.s002.pdf]

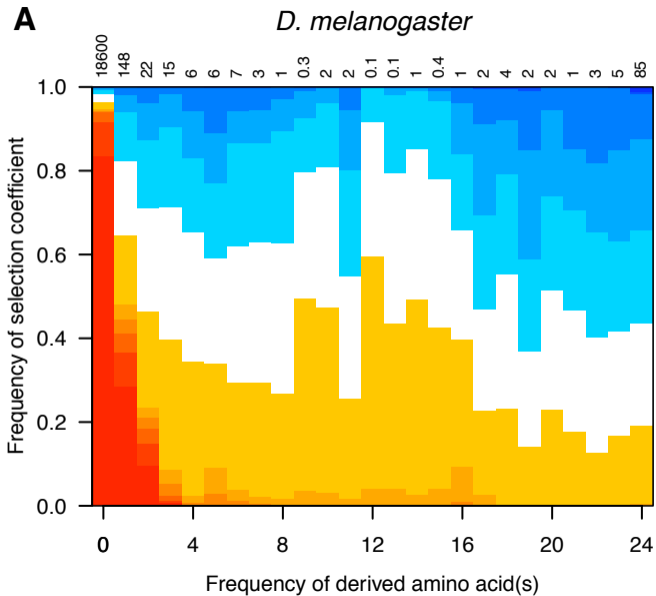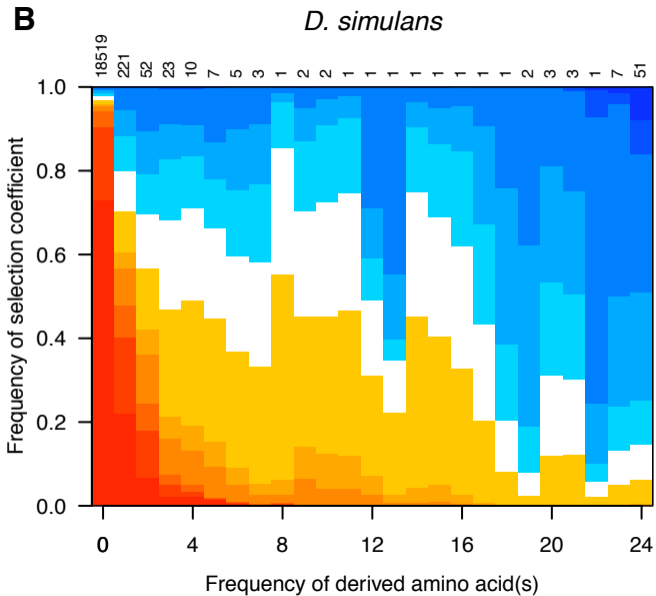

Supplement: Figure S3 — The distribution of fitness effects as a function of derived amino acid frequency in (A) D. melanogaster and (B) D. simulans. The frequency of selection coefficients was calculated in each category of sites, defined as the frequency of derived amino acids assuming a sample size of n = 24. Sites with n>24 were allocated to categories by resampling according to a hypergeometric distribution. Sites with n<24 were resampled according to binomial distribution. The vertical height of bars indicates the frequency of selection coefficients in that category, colored as in Figure 1. Above the barplot is printed the number of codons assigned to each category, averaged over the resampling. (PDF) [file pgen.1002395.s003.pdf]

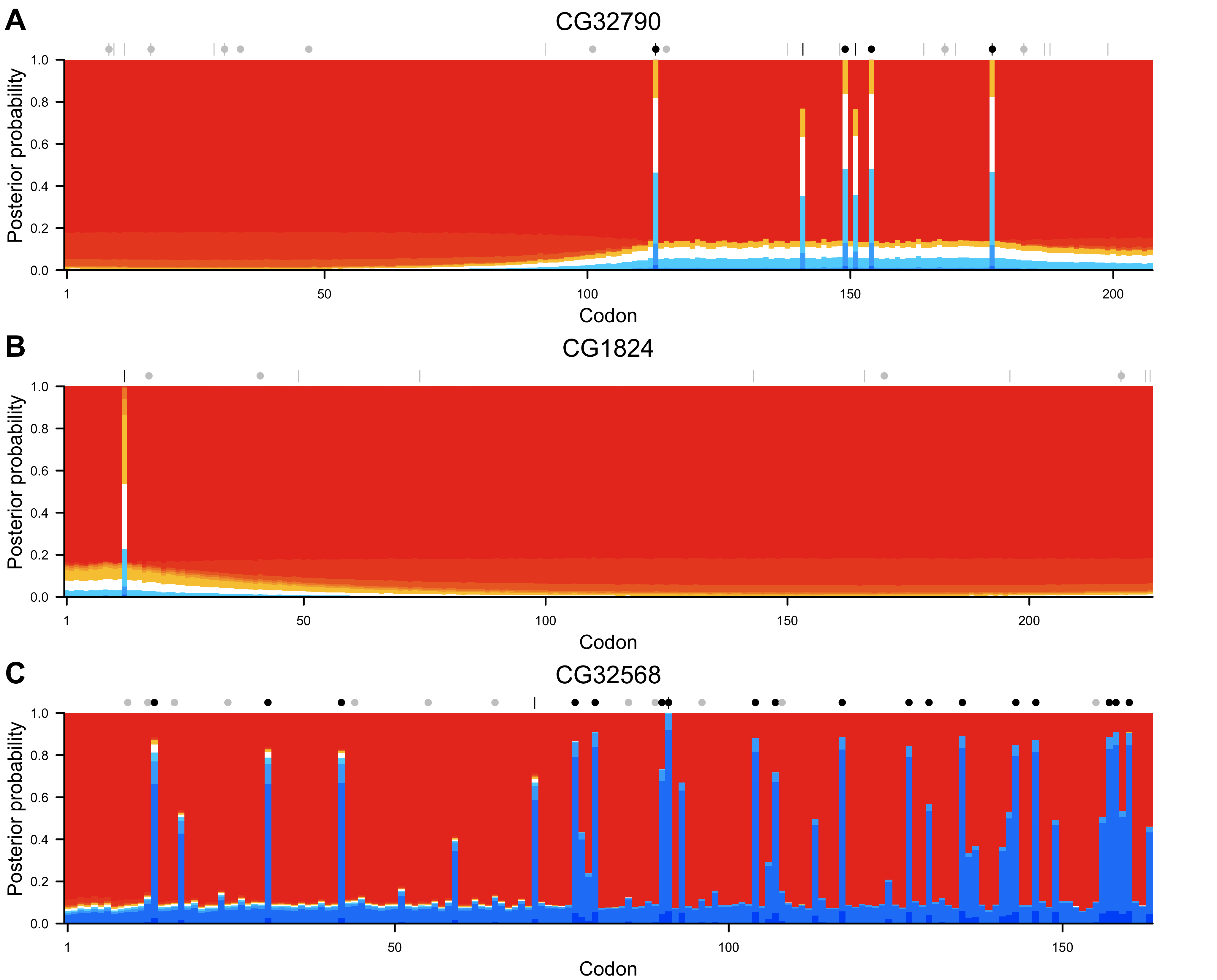

Supplement: Figure S4 — The posterior probability of selection coefficients for non-synonymous mutations along three genes in the D. melanogaster lineage. At each codon, the height of the colored bars represents the posterior probability of the corresponding selection coefficient, where colors closer to red represent increasingly deleterious variants, white represents neutral variants, and colors closer to blue represent increasingly beneficial variants, as in Figure 1. Above the barplot are indicated the presence of synonymous (grey) and non-synonymous (black) polymorphisms (vertical lines) and substitutions (circles) in the D. melanogaster lineage. (TIFF) [file pgen.1002395.s004.tif]

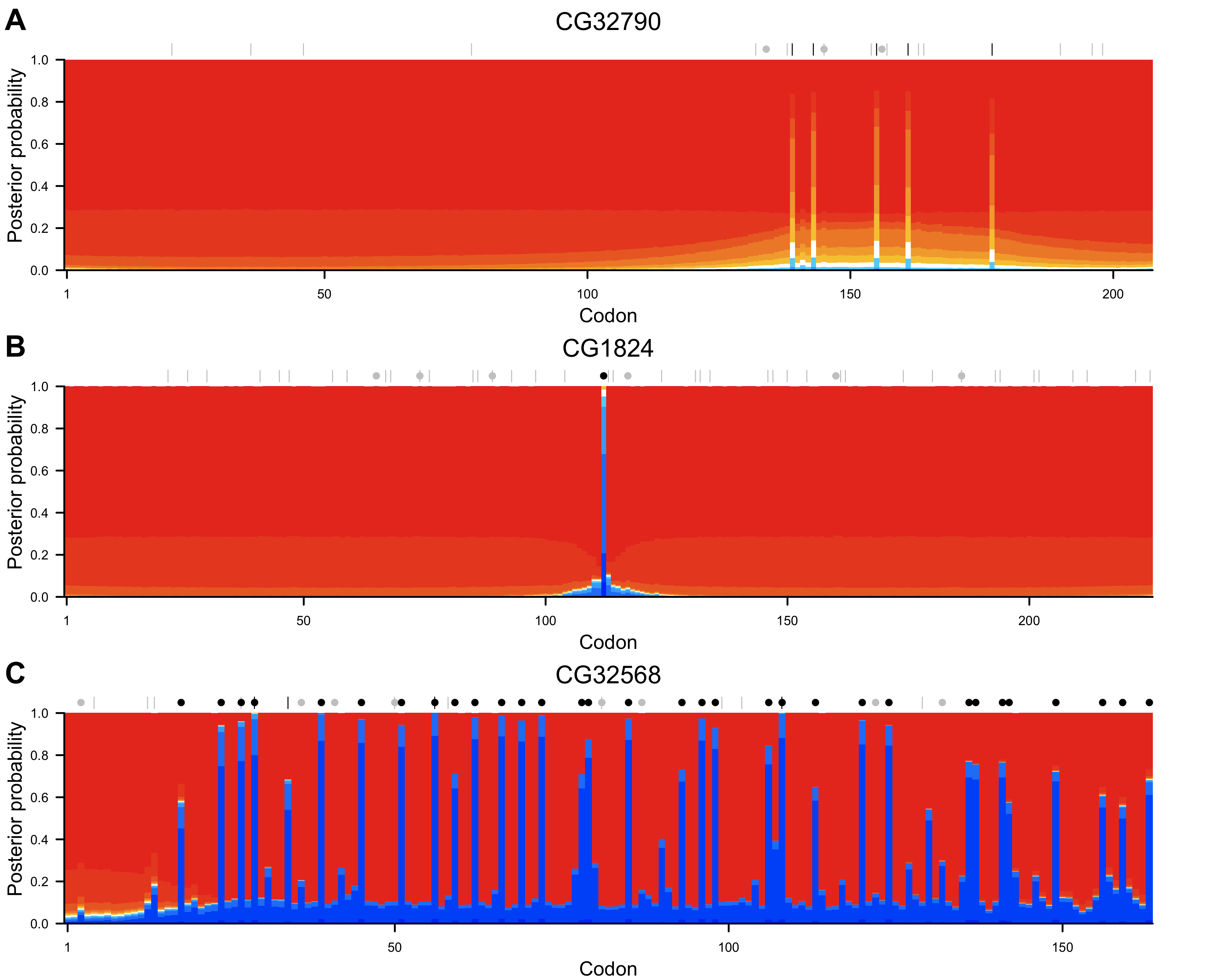

Supplement: Figure S5 — The posterior probability of selection coefficients for non-synonymous mutations along three genes in the D. simulans lineage. At each codon, the height of the colored bars represents the posterior probability of the corresponding selection coefficient, where colors closer to red represent increasingly deleterious variants, white represents neutral variants, and colors closer to blue represent increasingly beneficial variants, as in Figure 1. Above the barplot are indicated the presence of synonymous (grey) and non-synonymous (black) polymorphisms (vertical lines) and substitutions (circles) in the D. simulans lineage. (TIFF) [file pgen.1002395.s005.tif]

**A***D. melanogaster*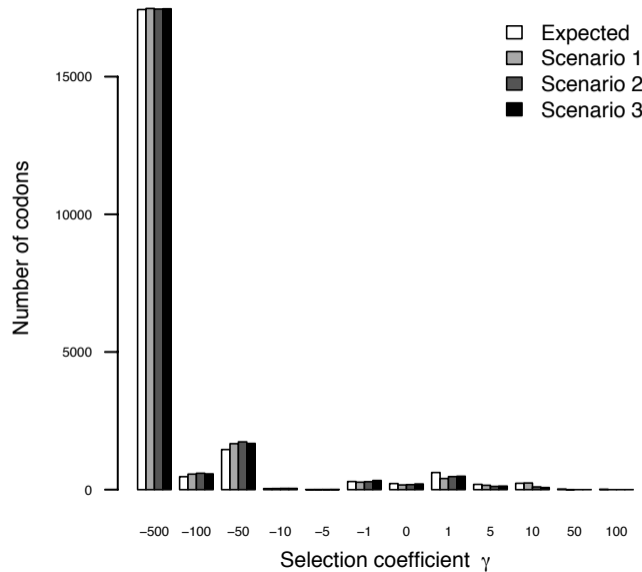**B***D. simulans*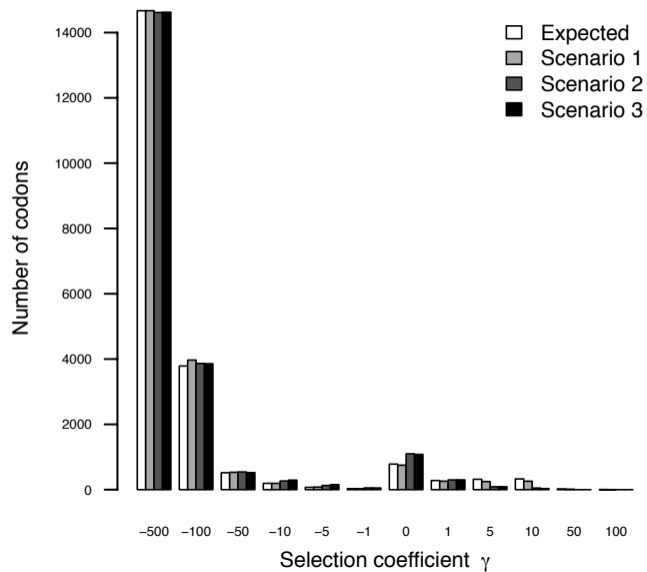

Supplement: Figure S6 — Robustness of inference of selection coefficients to linkage and demographic change. The frequency with which sites were assigned to each of the twelve selection classes is shown separately for (A) D. melanogaster and (B) D. simulans under three simulated scenarios assuming the DFE specified by the Expected column. Scenario 1: no linkage or demographic change. Scenario 2: demographic change but no linkage. Scenario 3: linkage and demographic change. (PDF) [file pgen.1002395.s006.pdf]

*D. melanogaster*

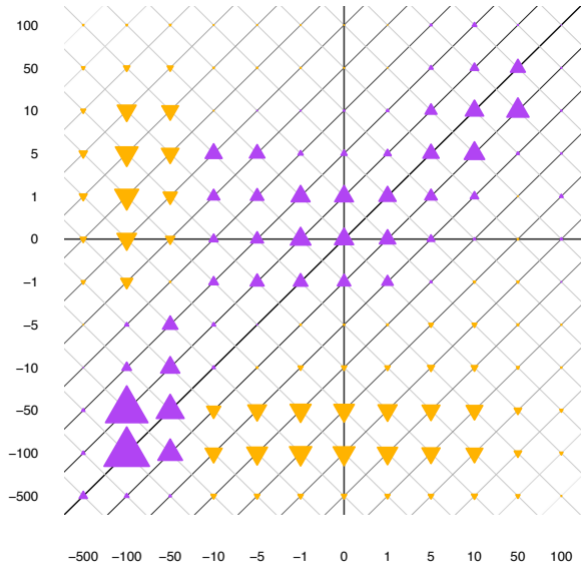

*D. simulans*

Supplement: Figure S7 — Correlation in the posterior probability of selection coefficients between D. melanogaster and D. simulans. For each pair of selection coefficients γmel and γsim, the magnitude of the correlation in posterior probability across sites is indicated by the size of the triangle and the direction by its colour: purple for positive values, orange for negative values. Positive correlations indicate an excess of sites compared to the assumption of independence between lineages. Negative correlations indicate a deficit of sites. In the top right and bottom left quadrants, γmel and γsim are concordant (both positive or both negative respectively). These quadrants are bisected by the diagonal, which indicates trends in the strength of selection. Between the diagonal and the horizontal line at γmel = 0, selection is stronger in D. simulans. Between the diagonal and the vertical line at γsim = 0, selection is weaker in D. simulans. In the other two quadrants γmel and γsim are discordant. (PDF) [file pgen.1002395.s007.pdf]

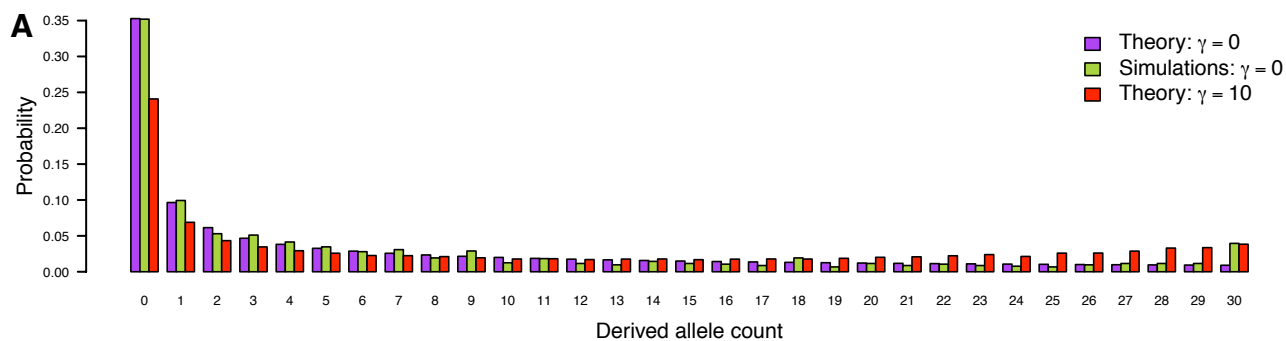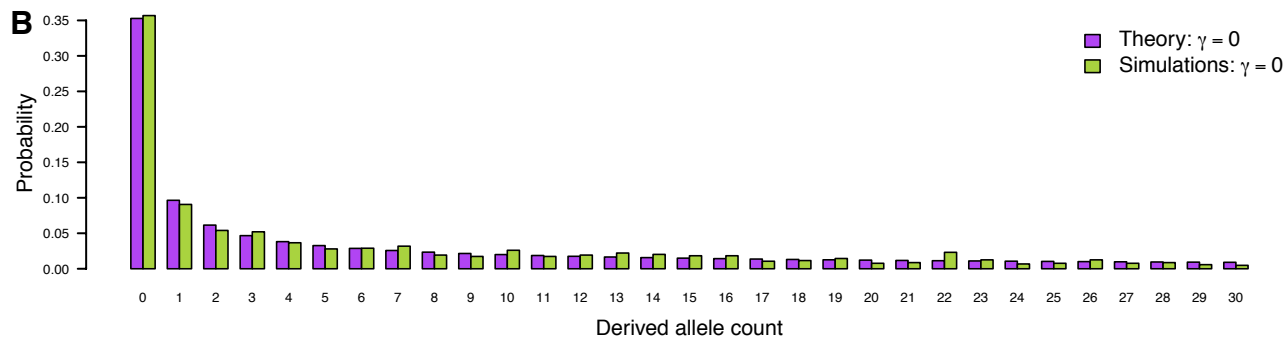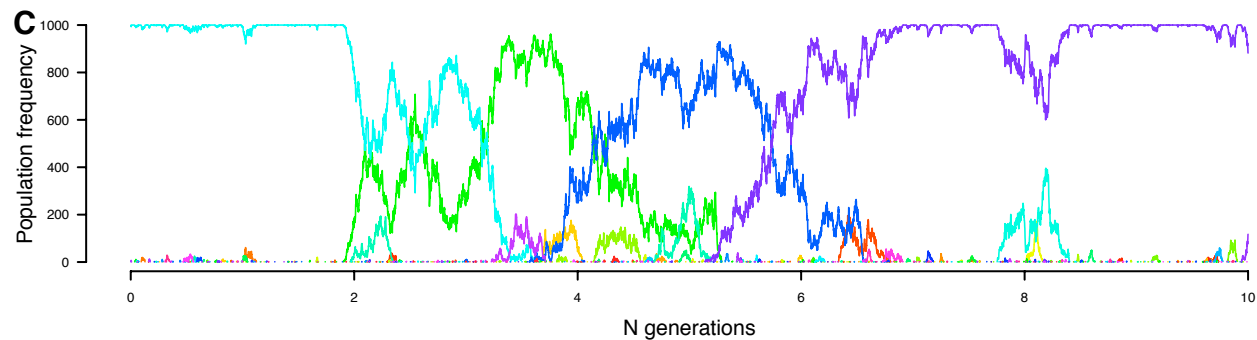

Supplement: Figure S8 — The operational definition of ancestral identity affects the accuracy of the conditional gene frequency distribution. (A) When the operational definition of ancestral identity is the last allele to have fixed or – as here – the state of the population MRCA, there is a discrepancy between theory (purple bars) and simulations (green bars). Simulations, which were conducted under the codon model with θ = 0.3, κ = 1 and γ = 0, are in agreement with theory when the ancestral allele is common, but report an elevated probability of not sampling the ancestral allele at all, which is not predicted from theory, and could be erroneously attributed to positive selection (red bars). (B) When the operational definition of ancestral identity is the oldest allele segregating in the population, the differences are resolved. (C) The cause of the problem: an ancestral allele (cyan) is lost from the population at 3.6 N generations, long before one of the other alleles (purple) fixes at 6.9 N generations, creating appreciable periods of time when the ancestral allele is no longer segregating in the population. (PDF) [file pgen.1002395.s008.pdf]

**A**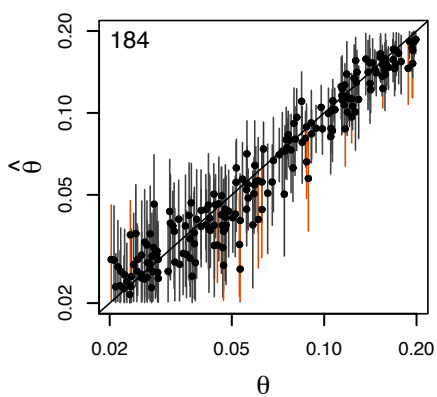

Population Genetic Model

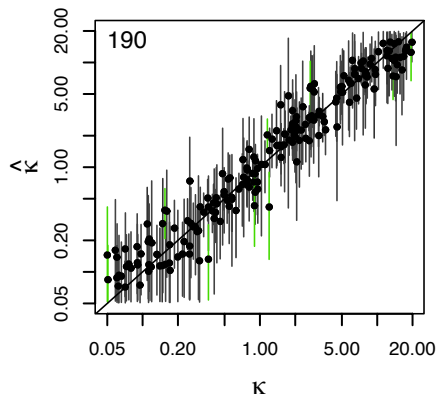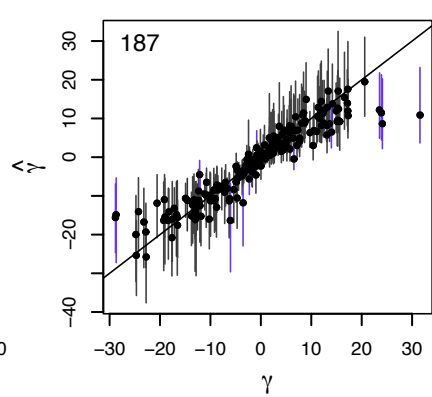**B**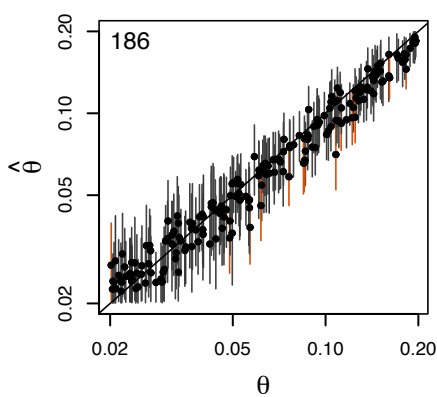

Population and Phylogenetic Model

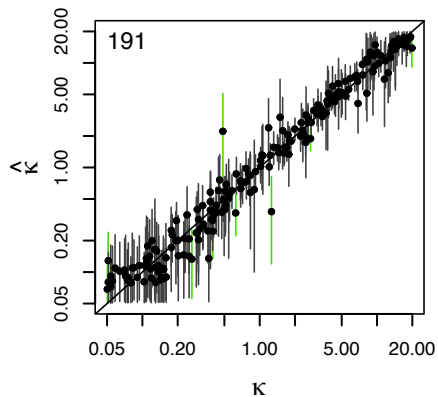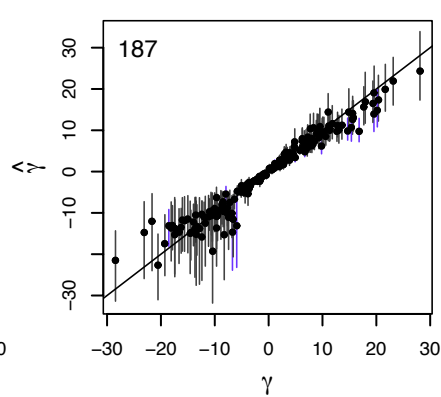

Supplement: Figure S9 — Testing the multiallelic codon model by simulation. The posterior mean (circles) and 95% credible interval (vertical lines) of the mutation rate (θ), transition:transversion ratio (κ) and strength of selection (γ) are plotted against their true values for 200 simulated datasets under two scenarios. (A) To test the conditional allele frequency distribution (the population genetic model), inference was performed with known ancestral states. (B) To additionally test the phylogenetic model and the extended pruning algorithm, the ancestral state was recorded 10 PNe generations prior to sampling. Colored lines draw attention to datasets for which the truth lies outside the 95% credible interval. The top left number in each graph reports the number of simulations for which the 95% credible interval enveloped the truth (a range of 184–196 is desirable). In all cases 30 sequences of length 250 codons were simulated per dataset. (PDF) [file pgen.1002395.s009.pdf]
