# Supplementary material for: A Population Genetics-Phylogenetics Approach to Inferring Natural Selection in Coding Sequences
Source: PLoS Genet. 2011 Dec 1;7(12):e1002395. doi: 10.1371/journal.pgen.1002395 (PMC3228810; doi:10.1371/journal.pgen.1002395)
Supplement: Text S1 — Deriving the multiallelic hot-or-not model. (PDF) [file pgen.1002395.s010.pdf]

Text S1: Deriving the Multiallelic Hot-or-Not Model

The greatest difficulty in applying the Wright-Dirichlet distribution to data analysis is computing the normalization constant. Various methods are available, including importance sampling and Taylor series expansion [1]. In a multiallelic PIMS model of directional selection with no dominance, the Wright-Dirichlet distribution simplifies to

$$p(\mathbf{f}) \propto \prod_{i=1}^{K} \mathrm{e}^{\gamma_i f_i} f_i^{\theta_i - 1}, \tag{A1}$$

where γ_i is the population-scaled selection coefficient for allele *i*. The normalizing constant for this distribution is related to the characteristic function for a Dirichlet distribution with parameter $\boldsymbol{\theta}$. In addition to the methods mentioned above, it can be computed numerically using contour integration in the complex plane [2, 3]

$$\int_{\Phi_K} \prod_{i=1}^{K} e^{\gamma_i f_i} f_i^{\theta_i - 1} \,\mathrm{d}\mathbf{f} = \frac{\prod_{i=1}^{K} \Gamma(\theta_i)}{2\pi I} \int_{L} e^{t} \prod_{i=1}^{K} (t - \gamma_i)^{-\theta_i} \,\mathrm{d}t, \tag{A2}$$

where I represents the imaginary unit and Φ_K is a $(K-1)$-simplex. The contour L is a loop beginning and ending at $-\infty$, and encircling in the positive direction (anticlockwise when the *x* and *y* axes correspond to the real and imaginary lines respectively) all the finite singularities of the integrand (*i.e.* when $t = \gamma_i$). We have *Mathematica* code that performs this integration (available on request) but found it to be numerically unstable.

Under the hot-or-not model, however, we can use Equation A2 to show that

$$p(\mathbf{f}) = \frac{\mathrm{e}^{\gamma F} \prod_{i=1}^{K} f_i^{\theta_i - 1}}{\mathrm{B}(\boldsymbol{\theta})\, {}_1\mathrm{F}_1(\Theta_H, \Theta, \gamma)}, \tag{A3}$$

where γ is the population-scaled selective difference between fitness classes, F is the total frequency of alleles in the favored (hot) fitness class, Θ_H is the total mutation rate to alleles of that class and Θ is the total mutation rate for all alleles. $\mathrm{B}(\boldsymbol{\theta})$ is the beta function with vector argument, so that

$$\mathrm{B}(\boldsymbol{\theta}) = \prod_{i=1}^{K} \Gamma(\theta_i) \Big/ \Gamma\left(\sum_{i=1}^{K} \theta_i\right), \tag{A4}$$

and ${}_1\mathrm{F}_1(a,b,c)$ is the confluent hypergeometric function.

Let $\mathcal{H}$ be the set of favored alleles, and $\mathcal{N}$ the set of disfavored alleles. The numbers of alleles in the two classes are K_H and K_N. Define $\mathbf{h} = \{ f_i / F : i \in \mathcal{H} \}$, $\mathbf{g} = \{ f_i / (1-F) : i \in \mathcal{N} \}$, $\boldsymbol{\theta}^{(H)} = \{ \theta_i : i \in \mathcal{H} \}$ and $\boldsymbol{\theta}^{(N)} = \{ \theta_i : i \in \mathcal{N} \}$. By making the change of variables $(\mathbf{f}) \to (F, \mathbf{g}, \mathbf{h})$, Equation A3 can be factorized so that

$$\begin{aligned} p(F,\mathbf{g},\mathbf{h}) = & \left[\frac{\mathrm{e}^{\gamma F} F^{\Theta_H - 1} (1-F)^{\Theta_N - 1}}{\mathrm{B}(\Theta_H, \Theta_N)\, {}_1\mathrm{F}_1(\Theta_H, \Theta, \gamma)} \right] \\ & \times \left[\frac{1}{\mathrm{B}(\boldsymbol{\theta}^{(N)})} \prod_{i=1}^{K_N} g_i^{\theta_i^{(N)} - 1} \right] \left[\frac{1}{\mathrm{B}(\boldsymbol{\theta}^{(H)})} \prod_{i=1}^{K_H} h_i^{\theta_i^{(H)} - 1} \right], \end{aligned} \tag{A5}$$

where $\Theta_N = \Theta - \Theta_H$. This demonstrates that F follows a biallelic Wright-Dirichlet distribution with parameters (Θ_H, Θ_N, γ), and $\mathbf{g}$ and $\mathbf{h}$ follow independent Dirichlet distributions with parameters $\boldsymbol{\theta}^{(N)}$ and $\boldsymbol{\theta}^{(H)}$ respectively. The Dirichlet distribution arises from the neutral case, and this factorization suggests that in a hot-or-not model, evolution within class $\mathcal{H}$ or $\mathcal{N}$ can be characterized as neutral. This in turn supports our assumption (see main text) that, in the low-mutation limit, the probability of fixation of

allele A equals the fixation probability for the whole class multiplied by the neutral fixation probability for allele A within its class.

To condition the stationary distribution on identity of the ancestral allele, suppose allele A is ancestral. In a recurrent selection model, the ancestral allele is disfavored. Thus from Equations A5 and the form of Equation 11 where A is disfavored,

$$p(F,\mathbf{g},\mathbf{h} \mid A) = \frac{\left(1-\mathrm{e}^{-\gamma(1-F)}\right) F^{\Theta_H - 1}(1-F)^{\Theta_N - 1} g_A \prod_{i=1}^{K_N} g_i^{\theta_i^{(N)}-1} \prod_{i=1}^{K_H} h_i^{\theta_i^{(H)}-1}}{\int_0^1 \left(1-\mathrm{e}^{-\varphi(1-F)}\right)\varphi^{\Theta_H-1}(1-\varphi)^{\Theta_N-1} d\varphi \int_{\Phi_{K_N}} \zeta_A \prod_{i=1}^{K_N} \zeta_i^{\theta_i^{(N)}-1} d\zeta \int_{\Phi_{K_H}} \prod_{i=1}^{K_H} \eta_i^{\theta_i^{(H)}-1} d\eta}$$
$$= \frac{\left(1-\mathrm{e}^{-\gamma(1-F)}\right) F^{\Theta_H - 1}(1-F)^{\Theta_N - 1} g_A \prod_{i=1}^{K_N} g_i^{\theta_i^{(N)}-1} \prod_{i=1}^{K_H} h_i^{\theta_i^{(H)}-1}}{\mathrm{B}(\Theta_H,\Theta_N)\left[1-{}_1\mathrm{F}_1(\Theta_N,\Theta,-\gamma)\right]\frac{\theta_A}{\Theta_N}\mathrm{B}\left(\boldsymbol{\theta}^{(N)}\right)\mathrm{B}\left(\boldsymbol{\theta}^{(H)}\right)}. \quad \text{(A6)}$$

Reversing the change-of-variables,

$$p(\mathbf{f} \mid A) = \frac{f_A}{1-F}\frac{\Theta_N}{\theta_A}\frac{\left(1-\mathrm{e}^{-\gamma(1-F)}\right)\prod_{i=1}^{K} f_i^{\theta_i - 1}}{\mathrm{B}(\boldsymbol{\theta})\left[1-{}_1\mathrm{F}_1(\Theta_N,\Theta,-\gamma)\right]}. \quad \text{(A7)}$$

Assuming that alleles are sampled at random with replacement from the population, we can utilize the multinomial distribution to obtain the conditional likelihood for a sample of size n comprising x_i copies of allele i, given that the ancestral allele is A.

$$p(\mathbf{x} \mid A) = \int_{\Phi_K} \binom{n}{\mathbf{x}} \prod_{i=1}^{K} f_i^{x_i} p(\mathbf{f} \mid A)\,\mathrm{d}\mathbf{f}$$
$$= \binom{n}{\mathbf{x}} \frac{(x_A+\theta_A)\Theta_N}{\theta_A(X_N+\Theta_N)} \frac{\mathrm{B}(\mathbf{x}+\boldsymbol{\theta})}{\mathrm{B}(\boldsymbol{\theta})} \frac{\left[1-{}_1\mathrm{F}_1(X_N+\Theta_N, n+\Theta,-\gamma)\right]}{\left[1-{}_1\mathrm{F}_1(\Theta_N,\Theta,-\gamma)\right]}, \quad \text{(A8)}$$

where X_N is the number of alleles sampled in class $\mathcal{N}$ and $\binom{n}{\mathbf{x}} = n!\prod_{i=1}^{K}\frac{1}{x_i!}$. Note that Equations A7 and A8 are equivalent to Equations 12 and 13 in the main text, using the identities $F_A = 1 - F$, $X_A = X_N$, and $\Theta_A = \Theta_N$.

References

1. Donnelly P, Nordborg M, Joyce P (2001) Likelihoods and simulation methods for a class of nonneutral population genetics models. Genetics 159: 853-867.
2. Erdéyli A (1939) Integration of certain systems of linear partial differential equations of hypergeometric type. Proc Roy Soc Edin A 59: 224-241.
3. Phillips PCB (1988) The characteristic function of the Dirichlet and multivariate F distributions. Cowles Foundation Discussion Paper No. 865.
