# Supplementary material for: A Population Genetics-Phylogenetics Approach to Inferring Natural Selection in Coding Sequences
Source: PLoS Genet. 2011 Dec 1;7(12):e1002395. doi: 10.1371/journal.pgen.1002395 (PMC3228810; doi:10.1371/journal.pgen.1002395)
Supplement: Text S2 — Approximating parent-dependent mutation. (PDF) [file pgen.1002395.s011.pdf]

# Text S2: Approximating Parent-Dependent Mutation

In developing an approximation to parent-dependent mutation we use a neutral approximation to the probability of mutation from the ancestral allele, $A$, to every other allele. It is convenient to employ the neutral case as the basis for our approximation, because there is an explicit separation between the genealogical process and the mutational process that does not hold more generally. In the neutral case we can use the coalescent [1, 2] to calculate the probability of observing a mutation conditional on the ancestral allele for both the parent-dependent and parent-independent mutation models. Our approach is to find a parent-independent mutation rate vector $\boldsymbol{\theta}'$ that matches the parent-dependent probabilities of observing an individual with allele $B$ given the ancestral allele $A$.

Taking the neutral limit $(\gamma \to 0)$ of the Wright-Dirichlet distribution conditional on the identity $A$ of the ancestral allele (main text Equation 8) yields

$$p(\mathbf{f} \mid A) = \frac{\prod_{i=1}^{K} f_i^{\theta_i + u_{Ai} - 1}}{\mathrm{B}(\boldsymbol{\theta} + \mathbf{u}_A)}, \tag{B1}$$

*i.e.* a Dirichlet distribution with parameter $\boldsymbol{\theta} + \mathbf{u}_A$, where $u_{Ai}$ equals 1 if $i = A$ and 0 otherwise. This suggests that conditioning on the ancestral allele is probabilistically equivalent to conditioning on having observed allele $A$ in a sample of size 1. Using the standard neutral coalescent model, we can calculate the probability, $m_{AB}$, of observing an allele of type $B$ having already observed an allele of type $A$, for general mutation models as

$$m_{AB} = \int_0^{\infty} p_{AB}^{(2t)} \, \mathrm{e}^{-t} \, \mathrm{d}t \,, \tag{B2}$$

since $t$ follows an exponential distribution with rate 1 when time is measured in units of $PN_e$ generations [1]. For a parent-independent model where the rate of mutation to allele $i$ is $\theta_i/2$, the transition probability from allele $A$ to $B$ in time $t$ is

$$p_{AB}^{(t)} = \begin{cases} \mathrm{e}^{-\Theta t/2} + \dfrac{\theta_B}{\Theta}\left(1 - \mathrm{e}^{-\Theta t/2}\right) & \text{if } A = B \\ \dfrac{\theta_B}{\Theta}\left(1 - \mathrm{e}^{-\Theta t/2}\right) & \text{if } A \neq B, \end{cases} \tag{B3}$$

where $\Theta = \sum_{i=1}^{K} \theta_i$ , therefore

$$m_{AB} = \begin{cases} \dfrac{\theta_B + 1}{\Theta + 1} & \text{if } A = B \\ \dfrac{\theta_B}{\Theta + 1} & \text{if } A \neq B. \end{cases} \tag{B4}$$

For a parent-dependent, time-reversible model,

$$p_{AB}^{(t)} = \sum_{i=1}^{K} v_{Ai} \, \mathrm{e}^{d_i t} \, v_{iB}^{(-1)}, \tag{B5}$$

where $\mathbf{v}$ is a matrix of eigenvectors of the mutation rate matrix, $\mathbf{v}^{(-1)}$ is its inverse, and $\mathbf{d}$ is a vector of the corresponding eigenvalues, so

$$m_{AB} = \sum_{i=1}^{K} \frac{v_{Ai} v_{iB}^{(-1)}}{1 - 2d_i} \,. \tag{B6}$$

For a given ancestral allele $A$, we wish to find $\boldsymbol{\theta}'$ so that $m_{AB}$ ($B = 1 \ldots K$) is matched for the parent-independent and parent-dependent models. The solution to this problem satisfies

$$\theta'_B = \begin{cases} m_{AB}\left(\Theta' + 1\right) - 1 & \text{if } A = B \\ m_{AB}\left(\Theta' + 1\right) & \text{if } A \neq B, \end{cases} \tag{B7}$$

where the vector $\mathbf{m}_A$ is calculated from Equation B6 for a time-reversible model. However, there are only $K - 1$ degrees of freedom because $\mathbf{m}_A$ is constrained to sum to 1; therefore $\Theta'$ is not identifiable. We circumvent the problem by imposing the additional constraint that $\theta'_A = 0$, interpretable as no back mutation to the ancestral allele, which is consistent with the low-mutation limit for the fixation probability used to motivate our implementation of phylogenetic conditioning. This implies that

$$\Theta' = \frac{1 - m_{AA}}{m_{AA}}. \quad \text{(B8)}$$

## References


1. Kingman JFC (1982) On the genealogy of large populations. J Appl Prob 19A: 27-43.
2. Hudson RR (1983) Properties of a neutral allele model with intragenic recombination. Theor Popul Biol 23: 183-201.
