# Supplementary material for: A Population Genetics-Phylogenetics Approach to Inferring Natural Selection in Coding Sequences
Source: PLoS Genet. 2011 Dec 1;7(12):e1002395. doi: 10.1371/journal.pgen.1002395 (PMC3228810; doi:10.1371/journal.pgen.1002395)
Supplement: Text S3 — On the definition of allelic ancestry. (PDF) [file pgen.1002395.s012.pdf]

Text S3: On the Definition of Allelic Ancestry

We used Wright-Fisher simulations to test the adequacy of our approximation to the likelihood in a parent-dependent model with selection, as described in the Methods. During simulation we kept a record of the ancestral allele at every generation, which we initially defined as the last allele to have reached fixation. Preliminary results revealed a discrepancy between our theory and the simulations. The major departure from theoretical expectations was the frequency with which we drew samples in which the ancestral allele was completely absent. Figure S8A shows the distribution of the number of derived alleles in a sample of 30 in a neutrally evolving population from theory (purple bars) and simulation (green bars). For the parameters used, simulations suggested there should be a 5% probability of failing to sample the ancestral type. Troublingly, only in the case of positive selection (red bars) did theory predict an appreciable probability of not sampling the ancestral allele. That neutral simulations should generate a pattern that the method confuses with positive selection was potentially of great concern, because we might detect positive selection in its absence.

Visualization of simulations over time revealed the cause of the problem (Figure S8C). When there are multiple alleles, the ancestral allele can be lost many generations before any other allele fixes. During these periods samples do not contain the ancestral allele. By contrast, in the low-mutation limit, no more than two alleles ever co-segregate so the problem does not arise. From one perspective, the discrepancy stems from utilizing the low-mutation limit for the probability of fixation when conditioning on the identity of the ancestral allele. The problem becomes worse as the mutation rate increases, to the point where fixation rarely occurs, and the identity of the last allele to have fixed has little

relevance to the current state of the population. In fact, in a PIMS model when $\theta > 1$, there is a non-zero probability that no allele will fix in a finite number of generations [1].

The fact that fixation is not even theoretically well-defined for some values of θ, and the observation that theory and simulations diverge even for modest mutation rates, led us to re-evaluate what the appropriate definition of allelic ancestry might in fact be when mutation is non negligible. We considered three alternatives for the operational definition of allelic ancestry:

1. The last allele to have fixed.
2. The allelic identity of the population MRCA.
3. The oldest allele still segregating in the population.

Note that all definitions are equivalent in the low-mutation limit. We repeated simulations using the alternative definitions of allelic ancestry, keeping track of the allelic state of the population most recent common ancestor (MRCA) or oldest allele. This is more difficult to implement because the population genealogy must be recorded and updated (by branching and pruning) from one generation to the next during Wright-Fisher simulation.

Using the population MRCA guarantees that the ancestral allele is well-defined, and quantitatively alleviated the discrepancy between theory and simulations, but did not eliminate it. Only by defining the ancestral allele as the oldest allele still segregating in the population, a definition that follows from a consideration of the age-ordered Ewens sampling formula [2, 3], did we reconcile the simulations with the results expected from theory (Figure S8B). This leads us to reason that firstly, the low-mutation limit of the probability that an allele was the last to fix (equivalently, is the next to fix) is a good approximation to the probability that an allele is the oldest (will persist the longest) when

mutation is non negligible. Secondly the statistical dependency of the allele frequency distribution on the identity of the oldest allele is stronger than its dependency on the identity of the last allele to fix or the population MRCA. In this sense, the identity of the oldest allele characterizes the state of the population better than other definitions of allelic ancestry when mutation is non negligible. Given this operational definition, we might wish to re-interpret the phylogenetic substitution process in terms of a change in the oldest allele, rather than a change in the fixed allele.

It is worth noting that in recurrent selection models, the definition of allelic ancestry affects the evolutionary dynamics, because the selection regime changes upon a change in the ancestral allele. We do not seek to defend this aspect of the model but rather wish to point out that is a consequence of the general approach of measuring fitness relative to the ancestral allele.

References

1. Karlin S, Taylor HM (1981) A Second Course in Stochastic Processes. New York: Academic Press.
2. Griffiths RC (2003) The frequency spectrum of a mutation, and its age, in a general diffusion model. Theor Popul Biol 64: 241-251.
3. Griffiths RC, Lessard S (2005) Ewens' sampling formula and related formulae: combinatorial proofs, extensions to variable population size and applications to ages of alleles. Theor Popul Biol 68: 167-177.
