# Supplementary material for: A Population Genetics-Phylogenetics Approach to Inferring Natural Selection in Coding Sequences
Source: PLoS Genet. 2011 Dec 1;7(12):e1002395. doi: 10.1371/journal.pgen.1002395 (PMC3228810; doi:10.1371/journal.pgen.1002395)
Supplement: Text S4 — Testing the multiallelic model by simulation. (PDF) [file pgen.1002395.s013.pdf]

Text S4: Testing the Multiallelic Model by Simulation

Since our multiallelic model of selection relies on the approximation we developed for parent-dependent mutation and selection (PDMS), we wished to evaluate its performance in inference.

Two scenarios were of particular interest: in the first, the ancestral allele is assumed known for each site in the population. This serves to test the performance of inference based on polymorphism data alone, which relies on an approximation to the stationary distribution of allele frequencies under parent-dependent mutation. In the second, the ancestral allele is assumed known at each site for a diverged ancestor existing 10 PN_e generations prior to sampling. This serves to test the performance of inference based on polymorphism and divergence data, which relies on the extended pruning algorithm to connect the population genetic phylogenetic components of the model.

Methods

Using our multiallelic model of selection, we simulated parameters 200 times as follows: θ from a log-uniform distribution with range (0.02, 0.2), κ from a log-uniform distribution with range (0.05, 20), γ from a normal distribution with mean 0 and standard deviation 10. For each of the 200 draws of (θ,κ,γ) we used Wright-Fisher simulations to generate data comprising $n = 30$ sequences of length $L = 250$ codons; sites were simulated independently. We then performed inference using Markov chain Monte Carlo (MCMC) to obtain a posterior distribution on the parameters (θ,κ,γ) for each dataset using for priors the distributions from which the parameters had been simulate the

parameters. Under these conditions, if our likelihood approximation is adequate, we expect [1] that the 95% credible intervals for each parameter will contain the truth in 95% of simulations, with an acceptance range of 184-196 datasets.

Preliminary simulations revealed a sensitivity to the precise definition of which allele is ancestral. We investigated a number of operational definitions of the ancestral allele; full details are provided in Text S3. The outcome was to define the ancestral allele as the oldest allele currently segregating in the population.

Results

For all parameters in both scenarios, the actual number of datasets in which the 95% credible interval (CI) enveloped the truth lay within the acceptable range (Figure S9). Broadly speaking, there is greater statistical uncertainty in the parameter estimates (represented by the width of the credible intervals) in the first scenario in which inference was based on polymorphism data alone. In the second scenario, one might have expected a loss of information because the identity of the ancestor is provided 10 PN_e generations prior to sampling. However, this seems to have been outweighed by the additional information gained from the signal of divergence.

All three parameters, including the transition:transversion ratio κ, were well-estimated from the data, indicating that the approximation to parent-dependent mutation works well. The uncertainty in estimating θ is greater for lower values, presumably because there are fewer informative sites. In estimating γ, there appears to be good power to distinguish positive from negative selection. However, the credible intervals get wider, representing greater uncertainty, as one moves away from $\gamma = 0$, suggesting that, not

surprisingly, the sign of the selection coefficient (positive or negative) is easier to estimate than its magnitude. This pattern is asymmetrical in the second scenario, in which the uncertainty surrounding γ is smaller for positive than negative selection. This can also be explained by the number of informative sites: positive selection increases the number of divergent sites by accelerating the substitution of non-synonymous mutations while negative selection slows their substitution.

Colored vertical lines in Figure S9 highlight 95% CIs that did not include the true value. This allows one to visualize any systematic biases that may be present in the simulations. For κ and γ there does not appear to be a consistent pattern in the datasets for which the 95% CI excluded the truth. However for θ it appears that in most of these cases, the mutation rate was under-estimated. This suggests a slight downward bias, although the scatter of point estimates (solid circles) around the truth indicates that it is not severe. The overall picture, therefore, is encouraging.

References

1. Dawid AP (1982) The well-calibrated Bayesian. Journal of the American Statistical Association. 77: 605-610.
