# Supplementary material for: A Population Genetics-Phylogenetics Approach to Inferring Natural Selection in Coding Sequences
Source: PLoS Genet. 2011 Dec 1;7(12):e1002395. doi: 10.1371/journal.pgen.1002395 (PMC3228810; doi:10.1371/journal.pgen.1002395)
Supplement: Text S5 — Inference via Markov chain Monte Carlo. (PDF) [file pgen.1002395.s014.pdf]

Text S5: Inference via Markov Chain Monte Carlo

We employed Markov chain Monte Carlo (MCMC) to obtain a sample from the posterior distribution of parameters. This approach was necessitated by the large number of parameters, in particular the selection coefficient γ at every site. Our general strategy was to use standard Metropolis-Hastings updates [1] to explore the posterior distribution of the parameters shared by all loci, which include the branch lengths T, transition:transversion ratios κ, the mean μ and variance σ^2 of the distribution of mutation rates across loci, the sliding window smoothing parameter p and the distribution of fitness effects (DFE) which is specified by $\lambda = \{\lambda_1, \lambda_2\}$.

For updating the branch- and site-specific selection coefficients γ, at first we tried Metropolis-Hastings moves similar to those employed by omegaMap [2]. However, this approach was hindered by the strong correlation between γ and the DFE λ, which led to poor mixing in the MCMC. An alternative strategy involves the use of Gibbs updates [3] to directly sample from the conditional posterior of γ given the DFE. The hidden Markov model (HMM) [4] structure of our sliding window model makes this possible, if the codon sequences are known for internal nodes of the phylogeny. This suggests an approach in which the codon sequences are Gibbs-sampled directly from the conditional posterior given γ using the pruning algorithm (main text Equation 3), and the branch- and site-specific selection coefficients γ are Gibbs-sampled directly from the conditional posterior given the ancestral codon sequences using the forward-backward algorithm (see below).

However, for the analysis of 100 X-linked coding sequences in three *Drosophila* species, we adopted a strategy that exploits the simplified structure of the pruning algorithm likelihood calculation for an unrooted phylogeny with only three taxa. Instead of explicitly exploring the branch- and site-specific selection coefficients using MCMC, we implicitly sum over them in the HMM using the forward-backward algorithm, and use a Gibbs update to propose changes to the sequence of the internal node one codon at a time.

HMM likelihood calculation

We used the HMM structure of our sliding window model to sum over the number of windows, the boundaries of those windows, and the value of γ within each window. We do this independently for each of the three lineages, conditional on the codon sequence of the internal node in the unrooted phylogeny.

The hidden variable of the HMM, $H \in [1, \Gamma_1]$, records the value of γ taken by the window. For lineage k, which includes the contemporary population and the branch leading to it, the emission probability at site i is

$$\varepsilon_{iH} = \sum_{s_k=1}^{K} P_{s_0 s_k}^{(v_k)} \left[\Lambda_1 \Pr\left(\mathbf{x}_k \mid s_{k,} \gamma_H\right) + \sum_{H'=1}^{\Gamma_2} \lambda_{2H'} \Pr\left(\mathbf{x}_k \mid s_{k,} \gamma_{H'}\right) \right], \qquad \text{(E1)}$$

where s_0 is the codon at the internal node of the phylogeny, s_k is the codon ancestral to the contemporary population, v_k is the branch length and $\Pr\left(\mathbf{x}_k \mid s_{k,} \gamma\right)$ is the conditional likelihood of the observed sequences in population k. This is the same term as in Equation 2 of the main text, but with the notation expanded to emphasize the dependence

on the selection parameter. The transition probability of the hidden variable from one codon to the next is

$$\delta_{HH'} = \begin{cases} \left(1-p\right) + p\dfrac{\lambda_{1H'}}{\Lambda_1} & \text{if } H' = H \\ p\dfrac{\lambda_{1H'}}{\Lambda_1} & \text{if } H' \neq H. \end{cases} \tag{E2}$$

The joint likelihood across sites is calculated using the forward algorithm,

$$\begin{aligned} a_{iH} &= \left[\sum_{H'=1}^{\Gamma_1} \delta_{H'H} a_{(i-1)H'}\right]\varepsilon_{iH} \\ &= \left[\left(1-p\right)a_{(i-1)H} + p\frac{\lambda_{1H}}{\Lambda_1}\sum_{H'=1}^{\Gamma_1} a_{(i-1)H'}\right]\varepsilon_{iH} \end{aligned} \tag{E3}$$

and the backward algorithm,

$$\begin{aligned} b_{iH} &= \sum_{H'=1}^{\Gamma_1} \delta_{HH'}\varepsilon_{(i+1)H'}b_{(i+1)H'} \\ &= \left(1-p\right)\varepsilon_{(i+1)H}b_{(i+1)H} + p\sum_{H'=1}^{\Gamma_1}\frac{\lambda_{1H'}}{\Lambda_1}\varepsilon_{(i+1)H'}b_{(i+1)H'} \end{aligned} \tag{E4}$$

subject to the conditions $a_{1H} = \lambda_{1H}\varepsilon_{1H}/\Lambda_1$ and $b_{LH} = 1$, when L is the length of the locus in codons. For any site i, the joint likelihood across sites is calculated as $\sum_{H=1}^{\Gamma_1} a_{iH}b_{iH}$. Because the site-specific values of γ are summed over by the HMM, we had to use posterior decoding to produce a sample from γ for recorded iterations of the MCMC.

Proposal distributions

The following Metropolis-Hastings moves were used to propose updates to scalar parameters from the current value X to new value X'.

1. Uniform proposal: $X' = X + Z$, where $Z \sim \text{Uniform}(-w, w)$. Applied to parameters defined on the interval $(-\infty, +\infty)$.

2. Log-normal proposal: $\log(X') = \log(X) + Z$, where $Z \sim \text{Normal}(0, s^2)$. Applied to parameters defined on the interval $(0, +\infty)$.

3. Logit-uniform proposal: $\log\left(\frac{X'}{1-X'}\right) = \log\left(\frac{X}{1-X}\right) + Z$, where $Z \sim \text{Uniform}(-w, w)$. Applied to parameters defined on the interval $(0,1)$.

The following Metropolis-Hastings move was used to propose updates to the DFE from the current value λ to new value λ'.

4. Switch proposal: $\lambda' = \lambda$ except that elements *i* and *j* are switched, where *i* and *j* are drawn uniformly from the elements of λ.

The acceptance probabilities were calculated as usual for Metropolis-Hastings moves [1]. The parameters of the proposal distributions were selected by manually tuning the MCMC.

A Gibbs sampler [3] was used to update the codon sequence of the internal node in the phylogeny. A codon was selected at random to update, the posterior density conditional on all 61 alternative codons was calculated, and a replacement codon drawn according to those probabilities.

Computation

All computations were implemented in C++. The GNU Scientific Library [5] was used to compute some functions including the beta function and confluent

hypergeometric function utilized in likelihood calculations (main text Equation 8). To reduce the running time, we utilized parallel processing. A systematic sweep MCMC was employed which was convenient for keeping the parallel processes synchronized across processors, as the next MCMC move was always known ahead of time. The details of MCMC updates were communicated between processes using the message passing interface (MPICH2 [6]), including actions such as whether to accept or reject a Metropolis-Hastings move. The computational load was divided into processes by locus, two per process in such a way as to minimize the variance in total sequence length among them. Each process recorded the values of locus-specific parameters (*i.e.* θ and γ), while the master process was responsible for recording the values of parameters common to all loci.

In all cases we ran two chains of 2,000,000 iterations, recording parameters from the MCMC at intervals of 40 iterations. After removing a burn-in of 20,000 iterations, chains were compared for convergence and then merged to obtain final results. Unless otherwise stated, we quote the posterior mean for point estimates and the (2.5%, 97.5%) quantiles for 95% credible intervals.

References

1. Hastings WK (1970) Monte Carlo sampling methods using Markov chains and their applications. Biometrika 57: 97-109.
2. Wilson DJ, McVean G (2006) Estimating diversifying selection and functional constraint in the presence of recombination. Genetics 172: 1411-1425.

3. Geman S, Geman D (1984) Stochastic relaxation, Gibbs distributions, and the Bayesian restoration of images. IEEE Transactions on Pattern Analysis and Machine Intelligence 6: 721-741.
4. Durbin R, SR Eddy, A Krogh, G Mitchison (1999) Biological Sequence Analysis: Probabilistic Models of Proteins and Nucleic Acids. Cambridge University Press, Cambridge.
5. Galassi M, J Davies, J Theiler, B Gough, G Jungman, et al. (2009) GNU Scientific Library Reference Manual, Third Edition. Network Theory Ltd.
6. Gropp W, E Lusk, R Thakur (1999) Using MPI-2: Advanced Features of the Message-Passing Interface. MIT Press, Cambridge.
