# Supplementary material for: A Population Genetics-Phylogenetics Approach to Inferring Natural Selection in Coding Sequences
Source: PLoS Genet. 2011 Dec 1;7(12):e1002395. doi: 10.1371/journal.pgen.1002395 (PMC3228810; doi:10.1371/journal.pgen.1002395)
Supplement: Text S6 — Robustness to linkage and demographic change. (PDF) [file pgen.1002395.s015.pdf]

Text S6: Robustness to Linkage and Demographic Change

Linkage disequilibrium and demographic change are known to have shaped patterns of genetic diversity in *D. melanogaster* and *D. simulans* [1, 2]. Further, it is known that these processes can influence the inference of selection from allele frequency information [3-5]. Since we did not explicitly account for linkage or demographic change in our method, we investigated the effects of these forces through simulation [6]. We drew a single set of parameters from the joint posterior distribution and simulated replica datasets under three scenarios: (1) no linkage or demographic change, (2) demographic change but no linkage, (3) linkage and demographic change. In the case of demographic change, we used parameters estimated under a two-epoch demographic model [4] from short intron data in *D. melanogaster* and *D. simulans* [1]. In the case of linkage, we applied the recombination rate estimate of $\rho = 100$ per kb in Zimbabwean and Kenyan populations of *D. melanogaster* [2] to both lineages. All parameters were fixed at the values used for simulation except the selection coefficients, which were estimated using our method. For Scenario 1, we obtained very close concordance between the frequency at which selection coefficients were assigned to codons, and that expected under the DFE (Figure S6). When demographic change was introduced (Scenario 2), the concordance was barely reduced, with almost no change in *D. melanogaster*, and a slight increase in the frequency of neutral mutations accompanied by a slight decrease in the frequency of moderately beneficial mutations in *D. simulans*. The introduction of linkage (Scenario 3) had no discernible effect on the concordance beyond that caused by demographic change. While our simulation procedure probably underestimates the full discrepancy when all

parameters are estimated, including the DFE itself, the results suggest our model provides a good fit to the data. What bias exists appears to be in the direction of underestimating the frequency of moderately beneficial mutations, and these simulations indicate that the magnitude of the bias is modest.

Methods

To investigate the effects of linkage and demographic change on the frequency with which codons are assigned one of the twelve selection coefficients, we performed simulations using SFS_CODE [6], a flexible forward simulator that can generate sequence data under a Wright-Fisher model with selection, linkage and population size changes. For a fixed set of parameters, principal among which was the DFE, we simulated a replica dataset of polymorphism and divergence data for the 100 genes in *D. melanogaster*, *D. simulans* and *D. yakuba*. Fixing the parameters at the values used for simulation, we then applied our sliding window method to probabilistically assign selection coefficients to codons. Under this simulation scheme, the expected frequency of sites assigned to each of the twelve selection coefficients is given by the DFE. Deviations away from the expectation reveal the effect of linkage or demography on our inference of selection. Since we fixed all parameters except the assignment of selection coefficients to codons, deviations from the expected DFE will be smaller than if we inferred those parameters. However, the simulation of hundreds of replica datasets would be computationally prohibitive, not to mention the time it would take to perform the subsequent analyses.

We investigated three scenarios: (1) no linkage or demographic change, (2) demographic change but no linkage, (3) linkage and demographic change. In all three

scenarios, we simulated data using the same parameter values for the DFE λ, the locus-specific mutation rates θ, the branch lengths T, the transition:transversion ratios κ, the sliding window smoothing parameters p, and the codon-specific selection coefficients. These parameters were drawn from the joint posterior distribution for the real data, obtained from a single, randomly chosen iteration of the MCMC. In the case of demographic change, we took the parameters of the two-epoch model [4] estimated from the site frequency spectrum of short introns [1]: *D. melanogaster* was estimated to have undergone a ten-fold expansion 0.011 N_0 generations ago (where N_0 is the contemporary size), and *D. simulans* a six-fold expansion 0.149 N_0 generations ago. In the case of linkage, we applied the estimate of $\rho = 100$ per kb for Zimbabwean and Kenyan populations of *D. melanogaster* [2] to all three lineages.

Even in the case of no linkage, Hill-Robertson interference [7] caused by selection at unlinked sites can reduce diversity in a manner that can be modeled as a straightforward reduction in the effective population size. Since the effective size is an emergent property of the interaction between sites, and not something that can be easily specified beforehand, we were obliged to obtain the desired effective population size by iterating until we matched diversity levels to those observed directly in each population. We adjusted N_e by fixing the census size at $N = 2000$, and scaling the mutation rates, recombination rate and selection coefficients accordingly. This was done independently on a gene-by-gene basis under each scenario.

References

1. Andolfatto P, KM Wong, D Bachtrog (2011) Effective population size and the efficacy of selection on the X chromosomes of two closely related Drosophila species. Genome Biol Evol 3: 114-128.
2. Haddrill PR, KR Thornton, B Charlesworth, P Andolfatto (2005) Multilocus patterns on nucleotide variability and the demographic and selection history of Drosophila melanogaster populations. Genome Res 15: 790-799.
3. Boyko AR, Williamson SH, Indap AR, Degenhardt JD, Hernandez RD, et al. (2008) Assessing the evolutionary impact of amino acid mutations in the human genome. PLoS Genet 4: e1000083.
4. Keightley PD, Eyre-Walker A (2007) Joint inference of the distribution of fitness effects of deleterious mutations and population demography based on nucleotide polymorphism frequencies. Genetics 177: 2251-2261.
5. Eyre-Walker A, Keightley PD (2009) Estimating the rate of adaptive molecular evolution in the presence of slightly deleterious mutations and population size change. Mol Biol Evol 26: 2097-2108.
6. Hernandez RD (2008) A flexible forward simulator for populations subject to selection and demography. Bioinformatics 24: 2786-2787.
7. Hill WG, A Robertson (1966) The effect of linkage on limits to artificial selection. Genet Res 8: 269-294.
